# IL-6 Inhibitory Compounds from the Aerial Parts of *Piper attenuatum* and Their Anticancer Activities on Ovarian Cancer Cell Lines

**DOI:** 10.3390/molecules29132981

**Published:** 2024-06-23

**Authors:** Hye Jin Kim, Lee Kyung Kim, Anna Kim, Khin Myo Htwe, Tae-Hwe Heo, Kye Jung Shin, Hee Jung Kim, Kee Dong Yoon

**Affiliations:** 1 College of Pharmacy, Integrated Research Institute of Pharmaceutical Sciences, The Catholic University of Korea, Bucheon 14662, Republic of Korea; kkhj980316@catholic.ac.kr (H.J.K.); dkssk@naver.com (A.K.); kyejung@catholic.ac.kr (K.J.S.); 2 College of Pharmacy, Integrated Research Institute of Pharmaceutical Sciences, BK21FOUR Team for Advanced Program for Smart Pharma Leaders, The Catholic University of Korea, Bucheon 14662, Republic of Korea; 1202dlruddl@catholic.ac.kr (L.K.K.); thhur92@catholic.ac.kr (T.-H.H.); 3 Popa Mountain National Park, Forest Department, Kyaukpadaung Township, Mandalay Division, Kyaukpadaung 05241, Myanmar; khinmyohtwe007@gmail.com

**Keywords:** *Piper attenuatum*, Piperaceae, maleimide-type alkaloid, piperimide I-III, IL-6 inhibition, (-)-loliolide, anticancer effects

## Abstract

*Piper attenuatum* Buch-Ham, a perennial woody vine belonging to the Piperaceae family, is traditionally used in Southeast Asia for treating various ailments such as malaria, headache, and hepatitis. This study described the isolation and identification of three new compounds, piperamides I-III (**1**–**3**), which belong to the maleimide-type alkaloid skeletons, along with fifteen known compounds (**4**–**18**) from the methanol extract of the aerial parts of *P. attnuatum*. Their chemical structures were elucidated using spectroscopic methods (UV, IR, ESI-Q-TOF-MS, and 1D/2D NMR). All the isolates were evaluated for their ability to inhibit IL-6 activity in the human embryonic kidney-Blue™ IL-6 cell line and their cytotoxic activity against ovarian cancer cell lines (SKOV3/SKOV3-TR) and chemotherapy-resistant variants (cisplatin-resistant A2780/paclitaxel-resistant SKOV3). The compounds **3**, **4**, **11**, **12**, **17**, and **18** exhibited IL-6 inhibition comparable to that of the positive control bazedoxifene. Notably, compound **12** displayed the most potent anticancer effect against all the tested cancer cell lines. These findings highlight the importance of researching the diverse activities of both known and newly discovered natural products to fully unlock their potential therapeutic benefits.

## 1. Introduction

*Piper attenuatum* Buch-Ham is a perennial woody vine belonging to the Piperaceae family and is mainly distributed in tropical Southeast Asian regions, such as India, Indonesia, Myanmar, and Laos. *Piper* is the largest genus within the family Piperaceae and encompasses over 700 species [1,2]. Renowned for its distinctive taste and scent, the *Piper* species holds widespread acclaim as a popular spice with considerable economic significance [3], and *P. attenuatum* is a plant called oval-leaved pepper, which can be used as an alternative to black pepper (*P. nigrum*) [4,5]. The leaves and roots of *P. attenuatum* are traditionally used to treat malaria, headache, hepatitis, mechanical injury, muscular pain, and pulmonary and urinary disorders [6,7,8].

The studies of the chemical composition of *P. attenuatum* have identified alkaloids, amides lignans, neolignans, and terpenes [7,9]. A few research groups have investigated the biological activities of *P. attenuatum* extracts, but there have been no systematic studies on the chemical identification of its biological activities. However, several studies have shown that *P. attenuatum* exhibits antibacterial, anticancer, antidiabetic, anti-inflammatory, antipyretic, diuretic, hepatoprotective, and hyperlipidemic activities [7].

Ovarian cancer is the eighth most common gynecological malignancy and the fifth most common cause of cancer-related deaths in women [10]. Interleukin (IL)-6 is a major immunoregulatory cytokine present in the ovarian cancer microenvironment [11]. It induces tumor angiogenesis, which plays an important role in the development of ascites and the metastasis of ovarian cancer, leading to rapid cancer progression and poor prognosis [12,13]. Preclinical evidence has shown that IL-6 improves ovarian cancer tumor cell survival and increases resistance to chemotherapy through the Janus kinase/signal transducer and the activator of transcription 3 (STAT3) signaling in tumor cells and the IL-6 receptor translocation in tumor endothelial cells [14]. IL-6 regulates some of its functions through the IL-6 receptor, which is a complex of IL-6Rα and glycoprotein (gp) 130 [11]. This makes the IL-6/IL-6Rα/gp130 signaling pathway an attractive target for therapeutic or preventive interventions. Currently, the application of IL-6/IL-6Rα/gp130 blockers as anticancer agents has not been extensively studied, especially for ovarian cancer.

**Figure 1:**
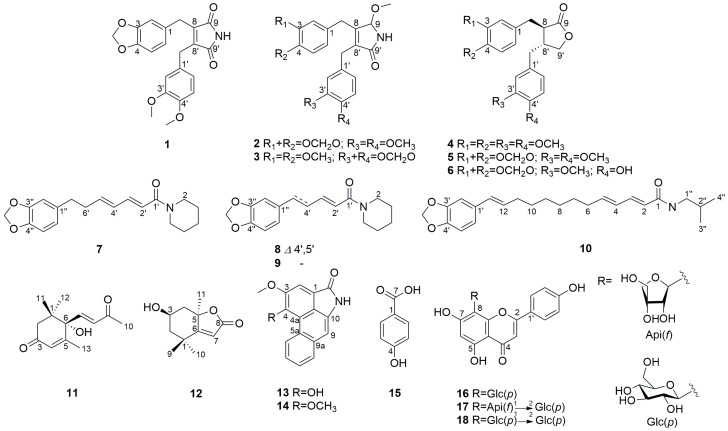
Chemical structures of compounds **1**–**18** from *P. attenuatum*.

In this study, we identified IL-6-targeting and -inhibitory compounds using isolates from *P. attenuatum* and evaluated their antitumor activity, particularly in ovarian cancer. The chemical investigation of *P. attenuatum* determined nine alkaloids (**1**–**3**, **7**–**10**, **13**–**14**), three lignans (**4**–**6**), a sesquiterpenoid (**11**), a monoterpenoid lactone (**12**), a simple phenolic compound (**15**), and three flavonoids (**16**–**18**) (Figure 1). Among the isolates, compounds **1**–**3** were previously undescribed, and six of the isolates exhibited IL-6 inhibitory activity and antitumor potential in human ovarian cancer cell lines. These findings suggest the potential of *P. attenuatum* as an effective resource against ovarian cancer and lay the groundwork for the development of novel therapeutic strategies against ovarian cancer.

## 2. Results and Discussion

### 2.1. Structure Elucidation

The dried and powdered *P. attenuatum* were extracted with MeOH and the concentrated extract was then partitioned with *n*-hexane, EtOAc, and *n*-BuOH. The EtOAc soluble fraction was resolved by various column chromatography on normal- and reversed-phase silica gel, Sephadex LH-20, and semi-preparative HPLC to yield compounds **1**–**18**.

**Figure 2:**
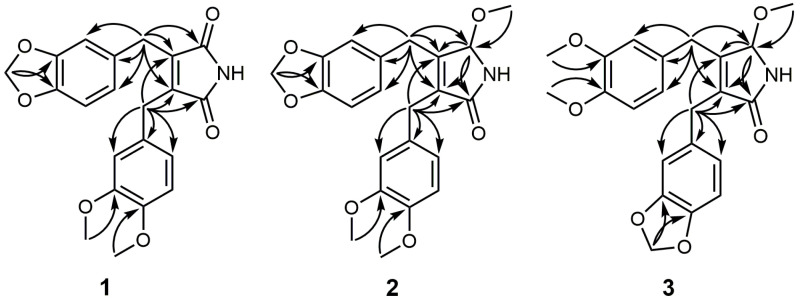
Key HMBC correlations of compounds **1**–**3**.

Compound **1** was obtained as a yellow amorphous powder, and its molecular formula was determined to be C_21_H_19_NO_6_ by ESI-Q-TOF-MS analysis, with an [M + Na]^+^ ion peak at *m*/*z* 404.1114. The IR spectrum exhibited strong bands at 3294, 1712, and 927 cm^−1^, indicating the presence of amine, imide, and methylenedioxy groups, respectively. It reacted positively with Dragendorff’s reagent, confirming the presence of alkaloids. The ^1^H-NMR spectrum displayed two sets of aromatic ABX-type resonances: one set at δ_H_ 6.69 (1H, d, *J* = 8.0 Hz, H-5), 6.61 (1H, d, *J* = 1.8 Hz, H-2), and 6.59 (1H, dd, *J* = 8.0, 1.8 Hz, H-6); the other set at δ_H_ 6.75 (1H, d, *J* = 8.0 Hz, H-5′), 6.67 (1H, dd, *J* = 8.0, 2.0 Hz, H-6′), and 6.64 (1H, d, *J* = 2.0 Hz, H-2′). Additionally, the spectrum revealed characteristic signals at δ_H_ 5.90 (2H, s, -OCH_2_O), indicating a methylenedioxy group; two methoxy groups at δ_H_ 3.83 (3H, s, 4′-OCH_3_) and 3.77 (3H, s, 3′-OCH_3_); and two methylene moieties at δ_H_ 3.66 (2H, s, H-7′) and 3.63 (2H, s, H-7). In the ^13^C-NMR spectrum, a total of 21 signals were observed, including two carbonyl groups at δ_C_ 171.4 (C-9′) and 171.3 (C-9). By comparing the observed ^13^C-NMR shift values with those reported in the literature, the presence of a substituted 2,5-pyrroledione skeleton, known as maleimide, was confirmed [15]. The analysis of the HMBC spectrum revealed significant correlations, as shown in Figure 2. The presence of the 3,4-methylenedioxybenzyl moiety was supported by the HMBC cross peaks of -OCH_2_O to C-3/C-4 and H-7 to C-1/C-2/C-6 and the existence of the 3′,4′-dimethoxybenzyl moiety is confirmed by the correlations of 3′-OCH_3_ with C-3′, 4′-OCH_3_ with C-4′, and H-7′ with C-1′/C-2′/C-6′. Moreover, the HMBC correlations from H-7 to C-8/C-8′/C-9 and H-7′ to C-8/C-8′/C-9′ firmly confirmed that **1** was a maleimide lignan, characterized by the linkage of two phenylpropane units through a β-β’ (8–8′) linkage. Based on spectroscopic evidence, the chemical structure of **1** was established, as shown in Figure 1, as piperimide I.

Compound **2** was obtained as a yellow amorphous powder and its molecular formula was established as C_22_H_23_NO_6_ based on the ESI-Q-TOF-MS analysis, which showed an [M + Na]^+^ peak at *m*/*z* 420.1423. The IR spectrum revealed the presence of an amine group (3340 cm^−1^), an amide group (1705 cm^−1^), and a methylenedioxy group (925 cm^−1^). Additionally, it reacted positively to Dragendorff’s reagent. The ^1^H- and ^13^C-NMR spectra of **1** and **2** revealed similarities (Table 1) with the presence of 3,4-methylenedioxybenzyl and 3′,4′-dimethoxybenzyl moiety. In the ^13^C-NMR spectrum of **2**, 22 signals were identified; the only difference was that the carbonyl group at the 9-position in **1** was replaced with a methoxy group in **2**. 

The HMBC spectrum supported the above information, with a cross peak from 9-OCH_3_ to C-9 and H-9 to C-9′/C-8/C-8′, which indicated the presence of a 3,4-disubstituted-5-methoxypyrrolin-2-one group (Figure 2). Additionally, the correlations between H-7 and C-8/C-9/C-8′, H-7′ and C-8/C-9′/C-8′ provide evidence that a 3,4-methylenedioxybenzyl group and a 3′,4′-dimethoxybenzyl group were connected to the positions at H-8 and H-8′, respectively (Figure 2). Based on the spectroscopic data, compound **2** was determined to be piperimide II.

Compound **3** was obtained as a yellow amorphous powder and its molecular formula was determined as C_22_H_23_NO_6_ by ESI-Q-TOF-MS at *m*/*z* 398.1603 [M + H]^+^. In the IR spectrum, the amine group (3390 cm^−1^), the amide group (1707 cm^−1^), and the methylenedioxy group (924 cm^−1^) were observed. A positive reaction was observed when tested using Dragendorff’s reagent. The 1D NMR spectrum of compound **3** was nearly identical to that of **2** (Table 1). The only difference between compounds **2** and **3**, as evidenced by the HMBC correlations (Figure 2), was that the 3′,4′-dimethoxybenzyl group was attached to C-8, and the 3,4-methylenedioxybenzyl group was linked to C-8′ in **3**. From the spectroscopic analysis, compound **3** was confirmed as piperimide III.

Compounds **2** and **3** were considered racemates, as indicated by their close-to-zero optical rotation values and the absence of Cotton effects in the CD spectrum. Consequently, the absolute configuration of C-9 in these compounds was not determined.

The main scaffold of **1**–**3** is maleimide, which possesses a five-membered heterocyclic structure belonging to the imide group and is called a lignan alkaloid. Maleimide is a natural compound mainly found in marine microorganisms and fungi [16] and its wide range of biological activities has prompted its synthesis based on structure–activity relationships (SARs) [17,18]. To date, maleimide derivatives have not been found in plants, and this study reports the first isolation and characterization of a maleimide derivative from a plant, thus expanding the natural sources of this significant structure.

However, compounds **1**–**3** represent structures with a maleimide moiety, a benzene ring bearing a methylenedioxy group, and fully methylated hydroxy groups. Interestingly, (-)-kusunokinin (**5**), a naturally occurring compound, shares structural similarities with compounds **1**–**3**, including the presence of a methylenedioxy group and fully methylated hydroxy groups [19,20]. While this suggests the potential natural origin of compounds **1**–**3**, the presence of methyl ether groups raises concerns about the possibility of artificial methylation during the extraction and isolation process. To definitively determine whether the methyl ether groups in compounds **1**–**3** are a result of natural occurrence or artificial methylation during the extraction and isolation process, further analysis using an alternative extraction solvent, such as ethanol instead of methanol, is warranted. This additional experiment will help elucidate the origin of the methyl ether groups and provide conclusive evidence regarding the natural or synthetic nature of these compounds.

The known compounds were determined to be (-)-dimethylmatairesinol (**4**) [21], (-)-kusunokinin (**5**) [22], (-)-haplomyrfolin (**6**) [23], piperdardine (**7**) [24], piperine (**8**) [25], piperanine (**9**) [25], guineensine (**10**) [25], (6*S*)-dehydrovomifoliol (**11**) [26], (-)-loliolide (**12**) [27], piperolactam A (**13**) [28], aristolactam BII (**14**) [29], p-hydroxybenzoic acid (**15**) [30], vitexin (**16**) [31], ficuflavoside (**17**) [32], and vitexin 2″-*O*-*β*-D-glucopyranoside (**18**) [33] by comparing their spectral data with that reported in the literature.

### 2.2. Screening of Compounds Using Human Embryonic Kidney (HEK)-Blue™ IL-6 Cells

The use of HEK-Blue reporter cell lines to screen for small-molecule drugs has recently gained popularity in cancer research [34,35]. We isolated eighteen compounds from *P. attenuatum* and assessed their inhibitory effects on IL-6 activity using the HEK-Blue™ IL-6 cell line. As the HEK-Blue™ IL-6 cells were transfected with a reporter gene expressing STAT3-inducible secreted embryonic alkaline phosphatase (SEAP), STAT3 was activated, and SEAP was produced when the cells were stimulated with IL-6. The extent of the inhibition of IL-6 downstream signaling by these compounds was assessed in vitro using bazedoxifene (gp130 inhibitor) as a positive control in the HEK-Blue™ IL-6 cells. Bazedoxifene is a newly developed third-generation indole-based estrogen receptor (ER) ligand used in the treatment of postmenopausal osteoporosis, also known as gp130 inhibitor. In our previous study, we demonstrated the anticancer effects of GP130 in ovarian cancer through combined administration of conventional anticancer drugs bazedoxifene and paclitaxel [36].

The HEK-Blue™ IL-6 cell bioassay showed that the eighteen isolates blocked IL-6-induced bioactivity, and six compounds (**3**, **4**, **11**, **12**, **17**, and **18**) inhibited IL-6 activity by approximately 50%, which was close to that of bazedoxifene (Figure S22). In addition, the degree of the inhibition of IL-6 activity according to the concentrations of bazedoxifene and the 16 isolates was compared in the HEK-Blue™ IL-6 cells (Figure S23). The cells were treated with increasing concentrations of the eighteen isolated compounds (0, 0.1, 1, 5, 10, 50, and 100 μM) in the presence of 10 ng/mL IL-6. The half-maximal effective concentration (EC_50_) of bazedoxifene was 1.905 ± 2.84 μM, and the therapeutic index (TI) value was 2.14. The EC_50_ values of compounds **3**, **4**, **11**, **12**, **17**, and **18** were 3.52 ± 0.14, 4.06 ± 0.07, 2.12 ± 0.04, 0.91 ± 0.04, 1.31 ± 0.05, and 0.579 ± 0.07 μM, respectively, and the TI values were >2 (Table 2). Compounds **12** and **18** displayed more potent IL-6 inhibitory activity than bazedoxifene at lower concentrations.

### 2.3. Anticancer Activity

Various chemotherapeutic drugs, particularly cisplatin and paclitaxel, have been used to treat ovarian cancer after surgical tumor removal. However, despite chemotherapy, many patients die of ovarian cancer recurrence. Inflammatory mediators such as IL-6 and several other cytokines influence drug resistance and recurrence in ovarian cancer [37]. The six compounds (**3**, **4**, **11**, **12**, **17**, and **18**) that inhibited IL-6 activity were screened for their antitumor effects in the A2780, SKOV3, cisplatin-resistant (A2780-cis), and paclitaxel-resistant (SKOV3-TR) ovarian cancer cell lines using a Cell Counting Kit-8 (CCK-8) assay. 

These compounds showed anti-proliferative activity in the ovarian cancer cell lines, but relatively weak activity in drug-resistant cell lines (Table 3). However, compounds **11** and **12** significantly reduced the viability of the A2780 and SKOV3 cells showing their half-maximal inhibitory concentration (IC_50_) values at around 10 µM. In addition, both the compounds showed more potent anti-proliferative activities against the A2780-cis and SKOV3-TR ovarian cancer cell lines than the positive control, bazedoxifene, indicating a stronger anticancer activity against the drug-resistant cancer cell lines. Compound **18** showed the most potent IL-6 inhibitory effect but exhibited weak anticancer activity. Upon treatment with compound **3**, maleimide derivative, the viability of the A2780 and SKOV3 cells was moderately inhibited with the IC_50_ values of 17.54 ± 0.19 and 15.76 ± 0.15 μM, respectively. 

Compound **12**, (-)-loliolide exhibited the most potent anticancer activity, comparable to that of bazedoxifene. (-)-Loliolide is a C11-terpene lactone, which arises from the breakdown of carotenoids by light or heat and is found in diverse marine algae and terrestrial plants [38]. In plants, (-)-loliolide serves as a defensive molecule against pathogens and herbivores and possesses allelopathic effects, such as germination and growth inhibition in other plants [39]. (-)-Loliolide has been shown to have several biological activities [40,41,42,43], but no studies on its anticancer effects have been reported. While research on the biological activities of *Piper* species has focused heavily on alkaloids, (-)-loliolide highlights the importance of investigating other secondary metabolites for potential benefits. Therefore, research on the diverse range of the activities of both known and newly discovered compounds is crucial to fully unveil the potential of natural products.

## 3. Materials and Methods 

### 3.1. General Experimental Procedures and Chemicals

The HPLC analyses were performed on an Agilent 1260 Infinity HPLC system (Agilent Technologies 6530, Santa Clara, CA, USA) with a 2695 pump and a 996 PDA detector. Preparative HPLC was performed using a Gilson HPLC system (Middleton, WI, USA) comprising a 321 pump, a UV/VIS-155 detector, and a fraction collector GX-271. The 1D- and 2D-NMR spectra were recorded using a Bruker AscendTM 500 spectrometer (Bruker, Karlsruhe, Germany). The MS data were obtained using an 6530 electrospray ionization quadrupole time-of-flight mass spectrometer (Agilent Technologies, Santa Clara, CA, USA). The UV spectra were measured using a Shimadzu UV-1880 spectrometer (Shimadzu USA Manufacturing Inc., Canby, OR, USA). The ATR-FTIR spectroscopy measurements were conducted using a Bruker TENSOR II FTIR spectrometer with a mile single-reflection horizontal ATR accessory (PIKE Technologies, Inc., Madison, WI, USA). The optical rotation was analyzed using a P-2000 polarimeter (Jasco, Tokyo, Japan). Silica gel 60 (40–63 μm, Merck, Darmstadt, Germany), octadecyl silica gel (40–63 μm, 230–400 mesh, Art. 9385, Merck, Darmstadt, Germany), and Sephadex LH-20 (25–100 μm, Amersham Pharmacia, Uppsala, Sweden) were used for column chromatography. The semi-preparative HPLC was performed with an ODS column (YMC-Pack-ODS-A, 250 × 20 mm ID, 5 μm, 120 Å, YMC, Tokyo, Japan) and the mobile phase consisted of methanol or acetonitrile (A) and water (B) using a gradient elution. The detection wavelength was 254 nm and the flow rate was 4.0 mL/min. The solvents for the medium-pressure liquid chromatography (MPLC) and high-performance liquid chromatography (HPLC) were purchased from Daejung Chemical & Metals (Gyunggido, Republic of Korea). Deionized water was purified using a Milli-Q water purification system (Millipore, Burlington, MA, USA). 

### 3.2. Plant Material

The aerial parts of *P. attenuatum* were collected from Popa Mountain National Park (Mandalay, Myanmar) in August 2011 and identified by Khin Myo Htwe (Popa Mountain National Park, Mandaley, Myanmar). A voucher specimen (#M-PA20110812) was deposited at the herbarium of the College of Pharmacy, the Catholic University of Korea. 

### 3.3. Extraction and Isolation

The dried aerial parts of *P. attenuatum* were stored in a freezer at –20 °C before use, and extraction, fractionation, compound isolation, and structural elucidation were carried out from December 2013 to August 2015. The isolated compounds had been kept in a deep freezer at –70 °C until the bioactivity screening which was performed from April to December 2023. Prior to the bioactivity screening, the purity of the isolated compounds was evaluated through the HPLC analysis using their relative peak area (%) (Tables S1 and S2). 

The dried and powdered sample material (450 g) was extracted with MeOH (2 L × 90 min × 3 times), and the extract was evaporated under reduced pressure to afford a MeOH extract (39 g). The extract was suspended in H_2_O (1.5 L) and fractionated by organic solvents (1.5 L × 3 times per each organic solvent) to yield *n*-hexane (12 g), EtOAc (16 g), and *n*-BuOH (3 g) soluble fractions. The EtOAc soluble fraction (16 g) was subjected to a silica gel column chromatography (C.C.) [CHCl_3_-MeOH (100:0 → 25:1, *v*/*v*, 2 L per solvent mixture)] to afford 9 sub-fractions (E1–E9). 

E1 was chromatographed on a Sephadex LH-20 column using MeOH to yield 12 subfractions (E1.1–E1.12). E1.7 was purified by RP-HPLC and eluted with MeOH-H_2_O (46:54, *v*/*v*) to yield compound **10** (25.2 mg, *t*_R_ = 46.4 min). E1.8 was subjected to RP-HPLC eluting with MeCN-H_2_O (55:45, *v*/*v*) to give subfraction E1.8.3, which was purified by RP-HPLC and eluted with MeOH-H_2_O (75:25, *v*/*v*) to yield compound **7** (18.6 mg, *t*_R_ = 37.2 min). E1.9 was subjected to RP-HPLC using 60% MeCN in H_2_O to obtain 6 subfractions (E1.9.1–E1.9.6). E1.9.4 yielded compound **9** (10.2 mg, *t*_R_ = 33.8 min). Compound **6** (6.7 mg, *t*_R_ = 31.9 min) was isolated from E1.9.2 by RP-HPLC and eluted with MeOH-H_2_O (70:30, *v*/*v*). The E1.9.3 was submitted to RP-HPLC with MeOH-H_2_O (74:26, *v*/*v*) to yield compound **1** (3.6 mg, *t*_R_ = 32.5 min). E1.9.6 was subjected to RP-HPLC using MeOH-H_2_O (65:35, *v*/*v*) to yield compound **5** (162 mg, *t*_R_ = 34.5 min). Compound **8** (7.7 mg, *t*_R_ = 34.1 min) was purified from E1.9.6.3 1 by RP-HPLC and eluted with MeOH-H_2_O (70:30, *v*/*v*). E1.11 was purified on a Sephadex LH-20 column and eluted with MeOH to obtain compound **4** (172 mg). E2 was subjected to chromatography over silica gel with CHCl_3_-MeOH (60:1, *v*/*v*) to yield 10 subfractions (E2.1–E2.10), and E2 was further subjected to Sephadex-LH20 column chromatography using MeOH to yield six subfractions (E2.1.1–E2.1.6). Compounds **11** (2.2 mg, *t*_R_ = 20.8 min) and **12** (2.9 mg, *t*_R_ = 21.4 min) were obtained from E2.1.2.5 using RP-HPLC with 46% MeOH in H_2_O. E2.1.5 was subjected to MPLC over silica gel and eluted with *n*-hexane-EtOAc (3:1, *v*/*v*) to yield six subfractions (E2.1.5.1–E2.1.5.6). Compounds **2** (3.7 mg, *t*_R_ = 31.0 min) and **3** (3.9 mg, *t*_R_ = 27.1 min) were isolated from E2.1.5.2 by RP-HPLC and eluted with MeOH-H_2_O (60:40, *v*/*v*). E2.1.6 was purified by RP-HPLC and eluted with MeOH-H_2_O (65:35, *v*/*v*) to obtain compounds **13** (2.1 mg, *t*_R_ = 29.0 min) and **14** (1.4 mg, *t*_R_ = 30.2 min). E7 was fractionated by chromatography on an RP-C18 column and eluted with MeCN-H_2_O (20:80–30:70–40:60, *v*/*v*) to obtain three fractions (E7.1–E7.3). Subsequently, E7.2 was separated using RP-HPLC with an isocratic solvent system of MeCN-H_2_O (25:75, *v*/*v*) to yield compounds **15** (1.6 mg, *t*_R_ = 16.1 min), **16** (2.0 mg, *t*_R_ = 18.5 min), **17** (2.7 mg, *t*_R_ = 17.9 min), and **18** (0.7 mg, *t*_R_ = 17.2 min).

### 3.4. Spectroscopic Data

Piperimide I (**1**): Yellow amorphous powder; ${\left[\alpha \right]}_{D}^{25}$−41.8 (*c* 0.1, MeOH); ESI-Q-TOF-MS: *m*/*z* 404.1114 (Calcd. for: 404.1110 [M + Na]^+^); UV (MeOH): *λ*_max_ (log*ε*) 283 (0.43), 223 (1.75), 206 (2.64) nm; IR (neat) *v*_max_ 3294, 2925, 2856, 1712, 1644, 1595, 1443, 1346, 1259, 1145, 1035, 927 cm^−1^; ^1^H-NMR (500 MHz, CDCl_3_) and ^13^C-NMR (125 MHz, CDCl_3_): see Table 1.

Piperimide II (**2**): Yellow amorphous powder; ${\left[\alpha \right]}_{D}^{25}$ 0.6 (*c* 0.03, MeOH); ESI-Q-TOF-MS: *m*/*z* 420.1423 (calcd for: 420.1423 [M + Na]^+^); UV (MeOH): *λ*_max_ (log*ε*) 285 (3.77), 205 (4.65); IR (neat) *v*_max_ 3340, 2926, 2853, 1705, 1591, 1514, 1489, 1441, 1364, 1247, 1139, 1034, 925, 861, 810, 765 cm^−1^; ^1^H-NMR (500 MHz, CDCl_3_) and ^13^C-NMR (125 MHz, CDCl_3_): see Table 1.

Piperimide III (**3**): Yellow amorphous powder; ${\left[\alpha \right]}_{D}^{25}$ −7.9 (*c* 0.03, MeOH); ESI-Q-TOF-MS: *m*/*z* 398.1603 (calcd for: 398.1604 [M + H]^+^); UV (MeOH): *λ*_max_ (log*ε*) 285 (3.89), 206 (4.63); IR (neat) *v*_max_ 3390, 2923, 2852, 1707, 1593, 1513, 1489, 1440, 1363, 1259, 1139, 1072, 1036, 924, 860, 810, 765 cm^−1^; ^1^H-NMR (500 MHz, CDCl_3_) and ^13^C-NMR (125 MHz, CDCl_3_): see Table 1.

### 3.5. Cell Culture

The human epithelial ovarian cancer cell lines SKOV3 and A2780 were purchased from the Korean Cell Line Bank (KCLB, Seoul, Republic of Korea) and the European Collection of Cell Cultures (ECACC, Wiltshire, UK), respectively. The cisplatin-resistant A2780 (A2780-cis) and paclitaxel-resistant SKOV3 (SKOV3-TR) cells were obtained from Yonsei University (Institute of Women’s Life Medical Science, Division of Gynecological Oncology, Republic of Korea). SKOV3, SKOV3-TR, A2780, and A2780-cis cells were cultured in the RPMI-1640 medium (Welgene, Daegu, Republic of Korea). SKOV3-TR and A2780-cis cells were cultured in 100 nM paclitaxel (Cayman Chemical Company, Ann Arbor, MI, USA) and 1 µM cisplatin (Dong-A Pharmaceutical Co., Seoul, Republic of Korea), respectively. All the culture media were supplemented with 10% (*v*/*v*) fetal bovine serum and 1% penicillin or streptomycin (Gibco-BRL, Gaithersburg, MD, USA), and the cells were maintained at 37 °C in a humidified atmosphere of 5% CO_2_ and 95% air. Each culture medium was replaced with a fresh medium every 2–3 days.

### 3.6. IL-6 Inhibition Bioassay Using HEK-Blue™ IL-6 Cells

The HEK-Blue™ IL-6 cells purchased from InvivoGen (San Diego, CA, USA) were used to seed 3 × 10^4^ cells/200 µL in a 96-well plate. Each well was treated with different concentrations (0, 0.1, 1, 5, 10, 50, and 100 μM) of the isolated compounds. Recombinant human IL-6 (BioLegend, San Diego, CA, USA) was administered at 10 ng/mL. After 24 h, 20 μL of the cell suspension was added to 180 μL of QUANTI-Blue™ solution (InvivoGen, San Diego, CA, USA) in a fresh 96-well plate. After incubation at 37 °C for 2 h, the absorbance was measured spectrophotometrically (Epoch, BioTek Instruments, Winooski, VT, USA) at 635 nm. The EC_50_ values were calculated from a log([drug]) versus normalized response curve fit using GraphPad Prism version 5.00 for Windows (GraphPad Software, San Diego, CA, USA). The CC_50_ values were calculated from extrapolated concentration–response curves plotted with GraphPad Prism version 5.0 for Windows (GraphPad Software, San Diego, CA, USA) using inhibitory percentages versus the drug concentration logarithm.

### 3.7. Cytotoxicity Assay

A CCK-8 assay was performed to assess cytotoxicity. After seeding 5 × 10^5^ cells/300 µL in 48-well plates, the cells were treated with the isolated compounds at 1, 5, 10, 20, 40, and 60 μM concentrations for 24 h. For the CCK-8 analysis, 30 μL of the CCK-8 solution (Dojindo, Kumamoto, Japan) was added to each well, allowed to react for 1 h, and then, the optical density value was measured at 450 nm using an ELISA microplate reader (Epoch, BioTek Instruments, Winooski, VT, USA). All the experiments were performed in triplicates. The IC_50_ values were calculated from a log([drug]) versus normalized response curve fit using GraphPad Prism version 5.00 for Windows (GraphPad Software, San Diego, CA, USA).

### 3.8. Statistical Analysis

All the graphs were constructed using the GraphPad Prism 8 software (GraphPad Software, San Diego, CA, USA). Statistical analyses were performed using one-way ANOVA with Tukey’s multiple comparison test and a Student’s *t*-test. The data are mean ± standard deviation (SD). Statistical significance was defined as *p* < 0.05. The levels of significance are indicated by * *p* < 0.05, ** *p* < 0.01, and *** *p* < 0.001.

## 4. Conclusions

In this study, we identified a total of eighteen compounds in the methanol extract of the aerial parts of *P. attenuatum*. Among these, three previously undescribed compounds **1**–**3**, piperamides I–III, have maleimide scaffolds. It was the first isolation of maleimide derivatives from plants, presenting a particularly intriguing discovery in phytochemistry. We evaluated the isolated compounds for their IL-6 inhibitory activity in the HEK-Blue IL-6 cell line. The six compounds exhibited IL-6 inhibition comparable to that of the positive control (bazedoxifene). Furthermore, we screened the IL-6 inhibitory compounds using an in vitro cytotoxicity test against the ovarian cancer cell lines (A2780/SKOV3) and drug-resistant variants (A2780-Cis/SKOV3-TR) to assess their anticancer potential. Among the isolates, (-)-loliolide (**12**) demonstrated the most potent anticancer activity against both the A2780 and SKOV3 cells, with the IC_50_ values of 8.62 ± 0.10 and 10.71 ± 0.31 μM, respectively, even exceeding the effects of bazedoxifene against drug-resistant cell lines. Although the ability of (-)-loliolide to inhibit IL-6 activity and its potential as an anticancer agent for ovarian cancer is promising, further research is necessary to elucidate the mechanisms underlying its anticancer effects. 

## Figures and Tables

**Table 1:** ^1^H- (CDCl_3_, 500 MHz) and ^13^C-NMR (CDCl_3_, 125 MHz) data of compounds **1–3**.

Position	1		2		3	
	δc	δ_H_ (Mult, *J* in Hz)	δc	δ_H_ (Mult, *J* in Hz)	δc	δ_H_ (Mult, *J* in Hz)
1	130.3		130.9		129.5	
2	109.5	6.61 (1H, d, 1.8)	109.5	6.50 (1H, d, 1.8)	112.0	6.49 (1H, d, 1.9)
3	148.2		148.1		149.1	
4	146.8		146.7		147.9	
5	108.7	6.69 (1H, d, 8.0)	108.6	6.67 (1H, d, 8.0)	111.4	6.75 (1H, overlap)
6	122.0	6.59 (1H, dd, 8.0, 1.8)	122.1	6.52 (1H, m)	121.0	6.64 (1H, dd, 8.1, 1.9)
7	29.3	3.63 (2H, s)	32.2	3.73 (1H, d, 13.6)	32.1	3.79 (1H, d, 14.9)
				3.33 (1H, d, 14.8)		3.38 (1H, d, 14.9)
8	141.1		152.6		152.8	
9	171.3		84.5	5.11 (1H, s)	84.3	5.09 (1H, s)
1′	129.0		131.4		132.5	
2′	112.3	6.64 (1H, d, 2.0)	111.6	6.75 (1H, overlap)	109.1	6.75 (1H, overlap)
3′	149.4		147.9		146.1	
4′	148.3		149.3		147.8	
5′	111.6	6.75 (1H, d, 8.0)	112.2	6.79 (1H, brs)	108.3	6.71 (1H, overlap)
6′	121.1	6.67 (1H, dd, 8.0, 2.0)	120.7	6.75 (1H, overlap)	121.4 (overlap)	6.71 (1H, overlap)
7′	29.4	3.66 (2H, s)	29.0	3.62 (2H, d, 6.5)	28.8	3.66 (1H, d, 14.8)
						3.58 (1H, d, 14.8)
8′	140.7		134.6		133.9	
9′	171.4		173.2		173.0	
3-OCH_3_					55.8	3.74 (3H, s)
4-OCH_3_					55.9	3.84 (3H, s)
3′-OCH_3_	56.1	3.77 (3H, s)	56.2	3.83 (3H, s)		
4′-OCH_3_	56.2	3.83 (3H, s)	56.1	3.80 (3H, s)		
9-OCH_3_			51.9	3.16 (3H, s)	51.9	3.18 (3H, s)
-OCH_2_O	101.3	5.90 (2H, s)	101.3	5.90 (2H, s)	100.9	5.89 (2H, s)

**Table 2:** IL-6 inhibitory activities for compounds **3**, **4**, **11**, **12**, **17**, and **18**.

Compound	EC_50_ (µM) ^a^	CC_50_ (µM) ^b^	TI ^c^
piperamide III (**3**)	3.52 ± 0.14	28.70 ± 0.19	8.16 ± 0.33
(-)-dimethylmatairesinol **(4**)	4.06 ± 0.07	22.64 ± 0.25	5.57 ± 0.32
(6*S*)-dehydrovomifoliol (**11**)	2.12 ± 0.04	13.72 ± 0.11	6.46 ± 0.15
(-)-loliolide (**12**)	0.91 ± 0.04	10.60 ± 0.10	11.58 ± 0.14
ficuflavoside (**17**)	1.31 ± 0.05	32.46 ± 0.45	24.77 ± 0.50
vitexin 2″-*O*-Glc (**18**)	0.58 ± 0.07	33.80 ± 0.45	58.35 ± 0.52
Bazedoxifene ^d^	1.91 ± 0.43	4.07 ± 0.35	2.14 ± 0.78

^a^ Half-maximal effective concentration (EC_50_) is the concentration of the drug at which effectiveness is inhibited by 50%; ^b^ 50% cytotoxic concentration (CC_50_) is the concentration that reduces the number of viable cells by 50% compared with the control; ^c^ therapeutic index was calculated as CC_50_/EC_50_; ^d^ positive control.

**Table 3:** Cytotoxicity of the compounds **3**, **4**, **11**, **12**, **17**, and **18** against human ovarian cancer cell lines.

Compound	IC_50_ (µM) ^a^			
	A2780	A2780-Cis	SKOV3	SKOV3-TR
piperamide III (**3**)	17.54 ± 0.19	40.51 ± 1.11	15.76 ± 0.15	40.98 ± 1.18
(-)-dimethylmatairesinol **(4**)	20.56 ± 0.25	16.95 ± 0.19	20.04 ± 0.18	33.01 ± 0.50
(6*S*)-dehydrovomifoliol (**11**)	9.80 ± 0.11	14.44 ± 0.21	10.69 ± 0.14	19.94 ± 0.22
(-)-loliolide (**12**)	8.62 ± 0.10	16.60 ± 0.18	10.71 ± 0.31	6.44 ± 0.10
ficuflavoside (**17**)	21.80 ± 0.45	41.04 ± 0.63	26.64 ± 0.30	40.34 ± 0.85
vitexin 2″-*O*-Glc (**18**)	40.97 ± 2.34	40.90 ± 3.07	23.07 ± 0.25	30.23 ± 0.48
Bazedoxifene ^b^	8.20 ± 1.77	33.80 ± 0.15	7.25 ± 0.62	20.32 ± 0.20

^a^ 50% inhibitory concentration; the values were expressed as the means (±SD) of logIC_50_; ^b^ positive control.

## Data Availability

The data presented in this study are available in the text and Supplementary Materials.

## Supplementary Materials

The following supporting information can be downloaded at: https://www.mdpi.com/article/10.3390/molecules29132981/s1, SI 1: Spectroscopic data of compounds **4**–**18**; Figure S1: ^1^H NMR spectrum (CDCl_3_, 500 MHz) of **1**; Figure S2: ^13^C NMR spectrum (CDCl_3_, 125 MHz) of **1**; Figure S3: HSQC spectrum of **1**; Figure S4: HMBC spectrum of **1**; Figure S5: ESI-Q-TOF-MS spectrum of **1**; Figure S6: UV spectrum of **1**; Figure S7: IR spectrum of **1**; Figure S8: ^1^H NMR spectrum (CDCl_3_, 500 MHz) of **2**; Figure S9: ^13^C NMR spectrum (CDCl_3_, 125 MHz) of **2**; Figure S10: HSQC spectrum of **2**; Figure S11: HMBC spectrum of **2**; Figure S12: ESI-Q-TOF-MS spectrum of **2**; Figure S13: UV spectrum of **2**; Figure S14: IR spectrum of **2**; Figure S15: ^1^H NMR spectrum (CDCl_3_, 500 MHz) of **3**; Figure S16: ^13^C NMR spectrum (CDCl_3_, 125 MHz) of **3**; Figure S17: HSQC spectrum of **3**; Figure S18: HMBC spectrum of **3**; Figure S19: ESI-Q-TOF-MS spectrum of **3**; Figure S20: UV spectrum of **3**; Figure S21: IR spectrum of **3**; Figure S22: Anti-IL-6 activities of the compounds **1**–**18** and bazedoxifene; Figure S23: Bioactivity test with compounds **1–18** in HEK-Blue™ IL-6 cells. Table S1: Analytical HPLC condition for compounds **1**–**18**; Table S2: HPLC chromatogram for compounds **1**–**18.**
