# Supplementary material for: IL-6 Inhibitory Compounds from the Aerial Parts of Piper attenuatum and Their Anticancer Activities on Ovarian Cancer Cell Lines
Source: Molecules. 2024 Jun 23;29(13):2981. doi: 10.3390/molecules29132981 (PMC11242996; doi:10.3390/molecules29132981)
Supplement: Supplementary file 1 [file molecules-29-02981-s001.zip › molecules-3053491-supplementary.pdf]

Supplementary Information

IL-6 inhibitory compounds from *Piper attenuatum* and their anticancer activities

Hye Jin Kim[1,†], Lee Kyung Kim[2,†], Anna Kim[1], Khin Myo Htwe[3], Tae-Hwe Heo[2], Kye Jung Shin[1], Hee Jung Kim[2,*], Kee Dong Yoon[1,*]

[1] College of Pharmacy and Integrated Research Institute of Pharmaceutical Sciences, The Catholic University of Korea, Bucheon 14662, Republic of Korea

[2] College of Pharmacy and Integrated Research Institute of Pharmaceutical Sciences, and BK21FOUR Team for Advanced Program for Smart Pharma Leaders, The Catholic University of Korea, Bucheon 14662, Republic of Korea

[3] Popa Mountain Park, Forest Department, Kyaukpadaung Township, Mandalay Division, Myanmar

† These authors contributed equally to this work.

Correspondence

[*] Kee Dong Yoon (kdyoon@catholic.ac.kr), College of Pharmacy, The Catholic University of Korea, 43 Jibong-ro, Bucheon 14662, Republic of Korea

[*] Hee Jung Kim (hjk0114@catholic.ac.kr), College of Pharmacy, The Catholic University of Korea, 43 Jibong-ro, Bucheon 14662, Republic of Korea

Contents

SI 1. Spectroscopic data of compounds **4 – 18** 2

Figure S1. ^{1}H NMR spectrum ($CDCl_3$, 500 MHz) of **1** 6

Figure S2. ^{13}C NMR spectrum ($CDCl_3$, 125 MHz) of **1** 6

Figure S3. HSQC spectrum of **1** 7

Figure S4. HMBC spectrum of **1** 7

Figure S5. ESI-Q-TOF-MS spectrum of **1** 8

Figure S6. UV spectrum of **1** 8

Figure S7. IR spectrum of **1** 9

Figure S8. ^{1}H NMR spectrum ($CDCl_3$, 500 MHz) of **2** 10

Figure S9. ^{13}C NMR spectrum ($CDCl_3$, 125 MHz) of **2** 10

Figure S10. HSQC spectrum of **2** 11

Figure S11. HMBC spectrum of **2** 11

Figure S12. ESI-Q-TOF-MS spectrum of **2** 12

Figure S13. UV spectrum of **2** 12

Figure S14. IR spectrum of **2** 13

Figure S15. ^{1}H NMR spectrum ($CDCl_3$, 500 MHz) of **3** 14

Figure S16. ^{13}C NMR spectrum ($CDCl_3$, 125 MHz) of **3** 14

Figure S17. HSQC spectrum of **3** 15

Figure S18. HMBC spectrum of **3** 15

Figure S19. ESI-Q-TOF-MS spectrum of **3** 16

Figure S20. UV spectrum of **3** 16

Figure S21. IR spectrum of **3** 17

Figure S22. Anti-IL-6 activities of the compounds **1-18** and bazedoxifene 17

Figure S23. Bioactivity test with compounds **1-18** in HEK-Blue™ IL-6 cells 18

Table S1. Analytical HPLC condition for compounds **1–18** 19

Table S2. HPLC chromatogram for compounds **1–18** 20

SI 1. Spectroscopic data of compounds **4-18**

methylarctigenin (**4**): yellowish amorphous powder; $C_{22}H_{14}O_4$; $[\alpha]^{25}_{D}$ -67.5 (*c* 0.04, $CHCl_3$); CD (EtOH) *Δε* (nm): +2.38 (211), -2.40 (232), +0.10 (285); Q-TOF-MS: *m/z* 409.1629 (Calcd for: 409.1657 $[M+Na]^+$); ^{1}H-NMR ($CDCl_3$, 500 MHz): δ 6.74 (2H, dd, *J* = 9.2, 8.1 Hz, H-5,5'), 6.66 (1H, d, *J* = 2.0 Hz, H-2), 6.63 (1H, dd, *J* = 8.0, 2.0 Hz, H-6), 6.53 (1H, dd, *J* = 8.1, 2.1 Hz, H-6'), 6.46 (1H, d, *J* = 2.1 Hz, H-2'), 4.09 (1H, m, H-9'a), 3.85 (1H, m, H-9'b), 3.83 (6H, d, *J* = 2.0 Hz, 3,4-OC$\underline{H}_3$), 3.81 (3H, s, 4'-OC$\underline{H}_3$), 3.80 (3H, s, 3'-OC$\underline{H}_3$), 2.92 (1H, dd, *J* = 10.6, 6.1 Hz, H-7), 2.65-2.43 (4H, m, H-7',8',8); ^{13}C-NMR ($CDCl_3$, 125 MHz): δ 178.9 (C-9), 149.3 (C-4), 149.2 (C-4'), 148.2 (C-3'), 148.1 (C-3), 130.7 (C-1), 130.4 (C-1'), 121.6 (C-6), 120.8 (C-6'), 112.6 (C-2), 112.1 (C-2'), 111.5 (C-5), 111.3 (C-5'), 71.4 (C-9'), 56.1-56.0 (3,4,3',4'-O$\underline{C}H_3$), 46.8 (C-8), 41.3 (C-8'), 38.4 (C-7'), 34.7 (C-7); ESI-Q-TOF-MS: *m/z* 409.1629 (Calcd for: 409.1657 $[M+Na]^+$).

(-)-kusunokinin (**5**): yellowish amorphous powder; $C_{21}H_{22}O_6$; $[\alpha]^{25}_{D}$ -46.1 (*c* 0.17, $CHCl_3$); CD (MeOH) *Δε* (nm): -3.38 (234), -0.20 (287); ^{1}H-NMR ($CDCl_3$, 500 MHz): δ 6.74 (1H, d, *J* = 8.0 Hz, H-5'), 6.69 (1H, d, *J* = 8.0 Hz, H-5), 6.58 (1H, brd, *J* = 2.0 Hz, H-2), 6.56 (1H, m, H-6'), 6.54 (1H, dd, *J* = 8.0, 2.0 Hz, H-6), 6.46 (1H, d, *J* = 2.0 Hz, H-2'), 5.91 (2H, dd, *J* = 5.0, 1.3 Hz, -OC$\underline{H}_2$O), 4.13 (1H, dd, *J* = 9.1, 7.0 Hz, H-9'a), 3.86 (1H, m, H-9'b), 3.83 (3H, s, 3'-OC$\underline{H}_3$), 3.80 (3H, s, 4'-OC$\underline{H}_3$), 2.94 (1H, dd, *J* = 14.1, 5.2 Hz, H-7a), 2.83 (1H, dd, *J* = 14.1, 7.1Hz, H-7b), 2.62-2.42 (4H, m, H-7'a,7'b,8,8'); ^{13}C-NMR ($CDCl_3$, 125 MHz): δ 178.7 (C-9), 149.3 (C-3'), 148.1 (C-4'), 148.0 (C-3), 146.7 (C-4), 131.6 (C-1), 130.6 (C-1'), 122.6 (C-6), 120.9 (C-6'), 111.9 (C-2'), 111.6 (C-5'), 109.7 (C-2), 108.4 (C-5), 101.3 (-O$\underline{C}H_2$O), 71.4 (C-9), 56.1 (4'-O$\underline{C}H_3$), 56.0 (3'-O$\underline{C}H_3$), 46.7 (C-8), 41.4 (C-8'), 38.5 (C-7'), 35.0 (C-7); ESI-Q-TOF-MS: *m/z* 393.1324 (Calcd for: 393.1314 $[M+Na]^+$).

(-)-haplomyrfolin (**6**): yellowish amorphous powder; $C_{20}H_{20}O_6$; $[\alpha]^{25}_{D}$ -68.5 (*c* 0.08, MeOH); CD (MeOH) *Δε* (nm): -1.14 (233); ^{1}H-NMR ($CDCl_3$, 500 MHz): δ 6.79 (1H, d, *J* = 8.0 Hz, H-5'), 6.70 (1H, d, *J* = 8.0 Hz, H-5), 6.59 (1H, d, *J* = 1.5 Hz, H-2), 6.57 (1H, dd, *J* = 8.0, 1.5 Hz, H-6), 6.50 (1H, dd, *J* = 8.0, 1.6 Hz, H-6'), 6.44 (1H, d, *J* = 1.6 Hz, H-2'), 5.91 (2H, dd, *J* = 3.5, 1.4 Hz, -OC$\underline{H}_2$O), 4.12 (1H, dd, *J* = 9.0, 1.2 Hz, H-9'a), 3.85 (1H, dd, *J* = 9.0, 7.4 Hz, H-9'b), 3.83 (3H, s, 3'-OC$\underline{H}_3$), 2.94 (1H, dd, *J* = 14.1, 5.1 Hz, H-7a), 2.83 (1H, dd, *J* = 14.1, 7.1 Hz, H-7b), 2.57 (1H, dd, *J* = 12.4, 5.0 Hz, H-7'a), 2.53 (1H, d, *J* = 6.0 Hz, H-8), 2.46 (1H, brd, *J* = 12.4 Hz, H-7'b), 2.45 (1H, dd, *J* = 10.0, 5.5 Hz, H-8'); ^{13}C-NMR ($CDCl_3$, 125 MHz): δ 178.7 (C-9), 148.2 (C-3), 146.8 (C-4), 146.7 (C-3'), 144.7 (C-4'), 131.6 (C-1), 130.0 (C-1'), 122.5 (C-6), 121.5 (C-6'), 114.7 (C-5'), 111.2 (C-2'), 109.7 (C-2), 108.4 (C-5), 101.2 (-O$\underline{C}H_2$O), 71.4 (C-9'), 56.1 (3'-O$\underline{C}H_3$), 46.7 (C-8), 41.5 (C-8'), 37.6 (C-7'), 35.0 (C-7); ESI-Q-TOF-MS: *m/z* 379.1162 (Calcd for: 379.1158 $[M+Na]^+$).

piperdardine (**7**): yellowish amorphous powder; $C_{19}H_{23}NO_3$; ^{1}H-NMR ($CDCl_3$, 500 MHz): δ 7.19 (1H, dd, *J* = 14.8, 10.8 Hz, H-3'), 6.69 (1H, d, *J* = 7.9 Hz, H-5''), 6.63 (1H, d, *J* = 1.6 Hz, H-2''), 6.58 (1H, dd, *J* = 7.9, 1.6 Hz, H-6''), 6.23 (1H, d, *J* = 14.8 Hz, H-2'), 6.16 (1H, dd, *J* = 15.3, 10.8 Hz, H-4'), 6.03 (1H, dt, *J* = 15.3, 7.4 Hz, H-5'), 5.89 (2H, s, -OC$\underline{H}_2$O), 3.58 (2H, brs, H-6), 3.45 (2H, brs, H-2), 2.63 (2H, t, *J* =8.7, 6.6 Hz, H-7'), 2.39 (2H, q, *J* = 7.4 Hz, H-6'), 1.65-1.58 (2H, m, H-4), 1.56-1.50 (4H, m, H-3,5); ^{13}C-NMR ($CDCl_3$, 125 MHz): δ 165.8 (C-1'), 147.8 (C-3''), 145.9 (C-4''), 142.7 (C-3'), 141.1 (C-5'), 135.4 (C-1''), 129.7 (C-4'), 121.4 (C-6''), 119.3 (C-2'), 109.0 (C-2''), 108.8 (C-5''), 101.0 (-O$\underline{C}H_2$O), 47.1 (C-6), 43.4 (C-2),

35.19 (C-6'), 35.16 (C-7'), 26.9 (C-5), 25.8 (C-3), 24.9 (C-4); ESI-Q-TOF-MS: *m/z* 314.1766 (Calcd for: 314.1756 $[M+H]^+$).

piperine (**8**): yellowish amorphous powder; $C_{17}H_{19}NO_3$; ^{1}H-NMR ($CDCl_3$, 500 MHz): δ 7.38 (1H, ddd, *J* = 14.7, 8.8, 1.4 Hz, H-3'), 6.96 (1H, d, *J* = 1.7 Hz, H-2''), 6.87 (1H, dd, *J* = 8.1, 1.7 Hz, H-6''), 6.76 (1H, dd, *J* = 8.1 Hz, H-5''), 6.72 (2H, m, H-4',5'), 6.42 (1H, d, *J* = 14.7 Hz, H-2'), 5.95 (2H, s, -OC$\underline{H}_2$O), 3.61 (2H, s, H-2), 3.50 (2H, s, H-6), 1.64 (2H, dt, *J* = 10.6, 3.5 Hz, H-4), 1.61-1.53 (4H, m, H-3,5) ; ^{13}C-NMR ($CDCl_3$, 125 MHz): δ 165.6 (C-1'), 148.4 (C-3''), 148.3 (C-4''), 142.7 (C-3'), 138.3 (C-5'), 131.3 (C-1''), 125.6 (C-4'), 122.7 (C-6''), 120.3 (C-2'), 108.7 (C-5''), 105.9 (C-2''), 101.4 (-O$\underline{C}H_2$O), 47.1 (C-6), 43.5 (C-2), 27.0 (C-5), 25.8 (C-3), 24.9 (C-4); ESI-Q-TOF-MS: *m/z* 286.1453 (Calcd for: 286.1443 $[M+H]^+$).

piperanine (**9**): yellowish amorphous powder; $C_{17}H_{19}NO_3$; ^{1}H-NMR ($CDCl_3$, 500 MHz): δ 6.77 (1H, dt, *J* = 15.0, 7.0 Hz, H-3'), 6.70 (1H, d, *J* = 7.9 Hz, H-5''), 6.65 (1H, d, *J* = 1.4 Hz, H-2''), 6.60 (1H, dd, *J* = 7.9, 1.4 Hz, H-6''), 6.19 (1H, d, *J* = 15.0 Hz, H-2'), 5.89 (2H, s, -OC$\underline{H}_2$O), 3.56 (2H, brs, H-2), 3.39 (2H, brs, H-6), 2.67 (2H, t, *J* = 7.4 Hz, H-5'), 2.44 (2H, q, *J* = 7.4 Hz, H-4'), 1.62 (2H, dt, *J* = 11.2, 5.6 Hz, H-4), 1.52 (4H, brs, H-3,5); ^{13}C-NMR ($CDCl_3$, 125 MHz): δ 165.7 (C-1'), 147.8 (C-3''), 146.0 (C-4''), 144.2 (C-3'), 135.2 (C-1''), 121.6 (C-2'), 121.4 (C-6''), 109.1 (C-2''), 108.4 (C-5''), 101.0 (-O$\underline{C}H_2$O), 47.1 (C-6), 43.3 (C-2), 34.8 (C-4'), 34.7 (C-5'), 26.8 (C-5), 25.8 (C-3), 24.8 (C-4); ESI-Q-TOF-MS: *m/z* 288.1597 (Calcd for: 288.1600 $[M+H]^+$).

guineensine (**10**): yellowish amorphous powder; $C_{17}H_{19}NO_3$; ^{1}H-NMR ($CDCl_3$, 500 MHz): δ 7.17 (1H, dd, *J* = 15.0, 10.3 Hz, H-3), 6.87 (1H, d, *J* = 1.4 Hz, H-2'), 6.73 (1H, dd, *J* = 7.9, 1.4 Hz, H-6'), 6.71 (1H, dd, *J* = 7.9 Hz, H-5'), 6.26 (1H, d, *J* = 15.7 Hz, H-13), 6.04 (2H, m, H-4,5), 6.02 (1H, d, *J* = 15.7 Hz, H-12), 5.90 (2H, s, -OC$\underline{H}_2$O), 5.73 (1H, d, *J* = 15.0 Hz, H-2), 3.14 (2H, t, *J* = 6.6 Hz, H-1''), 2.13 (4H, m, H-6,11), 1.78 (1H, dt, *J* = 13.4, 6.6 Hz, H-2''), 1.40 (4H, m, H-7,10), 1.30 (4H, m, H-8,9), 0.91 (3H, s, H-4''), 0.89 (3H, s, H-3''); ^{13}C-NMR ($CDCl_3$, 125 MHz): δ 166.6 (C-1), 148.1 (C-3'), 146.7 (C-4'), 143.3 (C-5), 141.5 (C-3), 132.7 (C-1'), 129.6 (C-13), 129.5 (C-12), 128.5 (C-4), 122.0 (C-2), 120.4 (C-6'), 108.4 (C-5'), 105.6 (C-2'), 101.1 (-O$\underline{C}H_2$O), 47.2 (C-1''), 33.1 (C-11), 33.0 (C-6), 29.6 (C-10), 29.2 (C-8), 29.1 (C-9), 28.9 (C-7), 28.8 (C-2''), 20.3 (C-3'',4''); ESI-Q-TOF-MS: *m/z* 406.2369 (Calcd for: 406.2358 $[M+H]^+$).

(6*S*)-dehydrovomifoliol (**11**): white amorphous powder; $C_{13}H_{18}O_3$; $[\alpha]^{28}_D$ +175 (*c* 0.03, MeOH); CD (MeOH) *Δε* (nm): +24.8 (258), -4.66 (322); ^{1}H-NMR (CD_3OD, 500 MHz): δ 6.92 (1H, d, *J* = 15.8 Hz, H-7), 6.37 (1H, d, *J* = 15.8 Hz, H-8), 5.87 (1H, m, *J* = 1.4 Hz, H-4), 2.53 (1H, d, *J* = 17.1 Hz, H-2a), 2.21 (1H, d, *J* = 17.1 Hz, H-2b), 2.24 (3H, s, H-10), 1.84 (3H, d, *J* = 1.4 Hz, H-13), 0.99 (3H, s, H-11), 0.95 (3H, s, H-12); ^{13}C-NMR (CD_3OD, 125 MHz) δ 200.8 (C-9), 200.5 (C-3), 164.8 (C-5), 148.5 (C-7), 131.9 (C-8), 128.2 (C-4), 80.1 (C-6), 50.7 (C-2), 42.8 (C-1), 27.8 (C-10), 24.9 (C-12), 23.7 (C-11), 19.3 (C-13); ESI-Q-TOF-MS: *m/z* 245.1158 (Calcd for: 245.1154 $[M+Na]^+$).

(-)-loliolide (**12**): white amorphous powder; $C_{11}H_{16}O_3$; $[\alpha]^{25}_D$ -36.8 (*c* 0.05, $CHCl_3$); CD (MeOH) *Δε* (nm): +1.75 (213), -14.82 (229); ^{1}H-NMR (CD_3OD, 500 MHz): δ 5.75 (1H, s, H-7), 4.21 (1H, p, *J* =3.6 Hz, H-3), 2.42 (1H, dt, *J* =13.3, 2.5 Hz, H-4a), 1.99 (1H, m, H-2a), 1.76 (3H, s, H-11), 1.74 (1H, d, *J* = 4.0 Hz, H-4b), 1.53 (1H, dd, *J* =14.5, 3.6 Hz, H-2b), 1.47 (3H, s, H-9), 1.28 (3H, s, H-10); ^{13}C-NMR (CD_3OD, 125 MHz): δ 185.9 (C-6), 174.6 (C-8), 113.5 (C-7), 89.1 (C-5), 67.4 (C-3), 48.1 (C-2), 46.6 (C-4), 37.3 (C-1), 31.2 (C-10), 27.6 (C-11), 27.1 (C-9); ESI-Q-TOF-MS: *m/z* 219.0998 (Calcd for: 219.0997 $[M+Na]^+$).

piperolactam A (**13**): white amorphous powder; $C_{16}H_{11}NO_3$; ^{1}H-NMR (DMSO-d_6, 500 MHz): δ 10.65 (1H, brs, -NH), 9.27 (1H, d, J = 6.1 Hz, H-5), 7.93 (1H, m, H-8), 7.76 (1H, s, H-2), 7.55 (1H, m, H-6), 7.54 (1H, m, H-7), 7.12 (1H, s, H-9), 4.05 (3H, s, 3-OCH3); ^{13}C-NMR (DMSO-d_6, 125 MHz): δ 168.9 (C=O), 149.4 (C-3), 148.2 (C-4), 135.2 (C-10), 134.1 (C-5a), 128.7 (C-8), 127.4 (C-5), 126.7 (C-9a), 126.6 (C-6), 124.9 (C-7), 124.3 (C-10a), 115.9 (C-1), 114.4 (C-4a), 108.5 (C-2), 104.3 (C-9), 57.2 (3-OCH3); ESI-Q-TOF-MS: *m/z* 288.0632 (Calcd for: 288.0637 $[M+Na]^+$).

aristolactam BII (**14**): pale yellow needles; $C_{17}H_{13}NO_3$; ^{1}H-NMR ($CDCl_3$, 500 MHz): δ 9.18 (1H, d, J = 8.0 Hz, H-5), 7.82 (1H, m, H-8), 7.64 (2H, m, H-6,7), 7.38 (1H, s, H-2), 7.18 (1H, s, H-9), 4.52 (3H, s, 3-OCH3), 4.05 (3H, s, 4-OCH3); ^{13}C-NMR ($CDCl_3$, 125 MHz): δ 153.2 (C-3), 110.5 (C-1), 64.0 (3-OCH3), 62.7 (4-OCH3); ESI-Q-TOF-MS: *m/z* 278.1479 (Calcd for: 278.0817 $[M+H]^-$).

p-hydroxybenzoic acid (**15**): white solid; $C_7H_6O_3$; ^{1}H-NMR (CD_3OD, 500 MHz): δ 7.87 (2H, d, J = 8.7 Hz, H-2,6), 6.81 (2H, d, J = 8.7 Hz, H-3,5); ^{13}C-NMR (CD_3OD, 125 MHz): δ 170.3 (C-7), 163.5 (C-4), 133.2 (C-2,6), 123.0 (C-1), 116.2 (C-3,5); ESI-Q-TOF-MS: *m/z* 139.0392 (Calcd for: 139.0395 $[M+H]^+$).

vitexin (**16**): yellowish amorphous powder; $C_{21}H_{20}O_{10}$; ^{1}H-NMR (DMSO-d_6, 500 MHz): δ 13.17 (1H, s, 5-OH), 10.86 (1H, s, 7-OH), 10.37 (1H, s, 4'-OH), 8.03 (2H, d, J = 8.8 Hz, H-2',6'), 6.89 (1H, d, J = 8.8 Hz, H-3',5'), 6.79 (1H, s, H-3), 6.27 (1H, s, H-6), 4.68 (1H, d, J = 9.9 Hz, H-1''), 3.83 (1H, m, H-2''), 3.76 (1H, dd, J = 11.4, 6.0 Hz, H-6''a), 3.52 (1H, dt, J = 11.4, 5.6 Hz, H-6''b), 3.38 (1H, dd, J = 9.6, 5.0 Hz, H-4''), 3.24 (2H, m, H-3'',5''); ^{13}C-NMR (DMSO-d_6, 125 MHz): δ 183.1 (C-4), 164.1 (C-2), 163.6 (C-5), 162.4 (C-7), 161.1 (C-4′), 156.0 (C-9), 129.1 (C-2′,6′), 121.4 (C-1′), 115.9 (C-3′,5′), 104.4 (C-8), 104.0 (C-10), 101.8 (C-3), 97.5 (C-6), 81.7 (C-5″), 78.3 (C-3″), 73.2 (C-1″), 70.2 (C-2″,4″), 60.8 (C-6″); ESI-Q-TOF-MS: *m/z* 433.1121 (Calcd for: 433.1135 $[M+H]^+$).

ficuflavoside (**17**): yellowish amorphous powder; $C_{26}H_{28}O_{14}$; ^{1}H-NMR (DMSO-d_6, 500 MHz): δ 13.17 (1H, s, 5-OH), 10.94 (1H, s, 7-OH), 10.37 (1H, s, 4'-OH), 8.04 (2H, d, J = 8.9 Hz, H-2',6'), 6.89 (2H, d, J = 8.9 Hz, H-3',5'), 6.78 (1H, s, H-3), 6.27 (1H, s, H-6), 5.00 (1H, d, J = 1.1 Hz, H-1'''), 4.72 (1H, d, J = 10.0 Hz, H-1''), 4.02 (1H, dd, J = 10.0, 8.5 Hz, H-2''), 3.77 (1H, m, H-6''a), 3.54 (1H, dd, J = 5.5, 1.1 Hz, H-2'''), 3.52 (1H, m, H-6''b), 3.41 (2H, m, H-3'',4''), 3.23 (1H, m, H-5''), 3.13 (1H, m, H-4'''a), 3.03 (1H, dd, J = 11.4, 5.5 Hz, H-4'''b), 2.94 (1H, d, J = 9.2 Hz, H-5'''a), 2.26 (1H, d, J = 9.2 Hz, H-5'''b); ^{13}C-NMR (DMSO-d_6, 125 MHz): δ 182.0 (C-4), 163.9 (C-2), 162.7 (C-5), 161.3 (C-7), 160.5 (C-4'), 155.3 (C-9), 129.0 (C-2',6'), 121.6 (C-1'), 115.8 (C-3',5'), 109.3 (C-1'''), 104.2 (C-8), 103.9 (C-10), 102.4 (C-3), 98.1 (C-6), 81.75 (C-5''), 79.07 (C-3''), 79.04 (C-3'''), 75.5 (C-2'''), 74.8 (C-2''), 73.2 (C-5'''), 71.7 (C-1''), 70.7 (C-4''), 64.2 (C-4'''), 61.1 (C-6''); ESI-Q-TOF-MS: *m/z* 565.1543 (Calcd for: 565.1557 $[M+H]^+$).

vitexin 2''-*O*-β-D-glucopyranoside (**18**): yellowish amorphous powder; $C_{27}H_{30}O_{15}$; ^{1}H-NMR (DMSO-d_6, 500 MHz): δ 13.15 (1H, s, 5-OH), 8.00 (2H, d, J = 8.8 Hz, H-2',6'), 6.89 (2H, d, J = 8.8 Hz, H-3',5'), 6.74 (1H, s, H-3), 6.22 (1H, s, H-6), 4.80 (1H, d, J = 10.0 Hz, H-1''), 3.92 (1H, d, J = 7.8 Hz, H-1'''); ^{13}C-NMR (DMSO-d_6, 125 MHz): δ 164.1 (C-2), 161.5 (C-4'), 160.7 (C-5), 156.4 (C-9), 128.0 (C-2',6'), 121.3 (C-1'), 115.4 (C-3',5'), 104.78 (C-1'''), 103.5 (C-8), 103.5 (C-10), 102.2 (C-3), 97.5 (C-6), 81.3 (C-5''), 80.9 (C-2''), 77.9 (C-3''), 75.8 (C-5'''), 75.8 (C-3'''), 73.6 (C-2'''), 71.1 (C-1''), 69.4 (C-4''), 68.9 (C-4'''), 60.8 (C-6''), 60.0 (C-6'''), ND (C-7, C-8, C-4) (ND=Not Detected); ESI-Q-TOF-MS: *m/z* 593.1531 (Calcd for: 593.1506 $[M+Na]^+$).

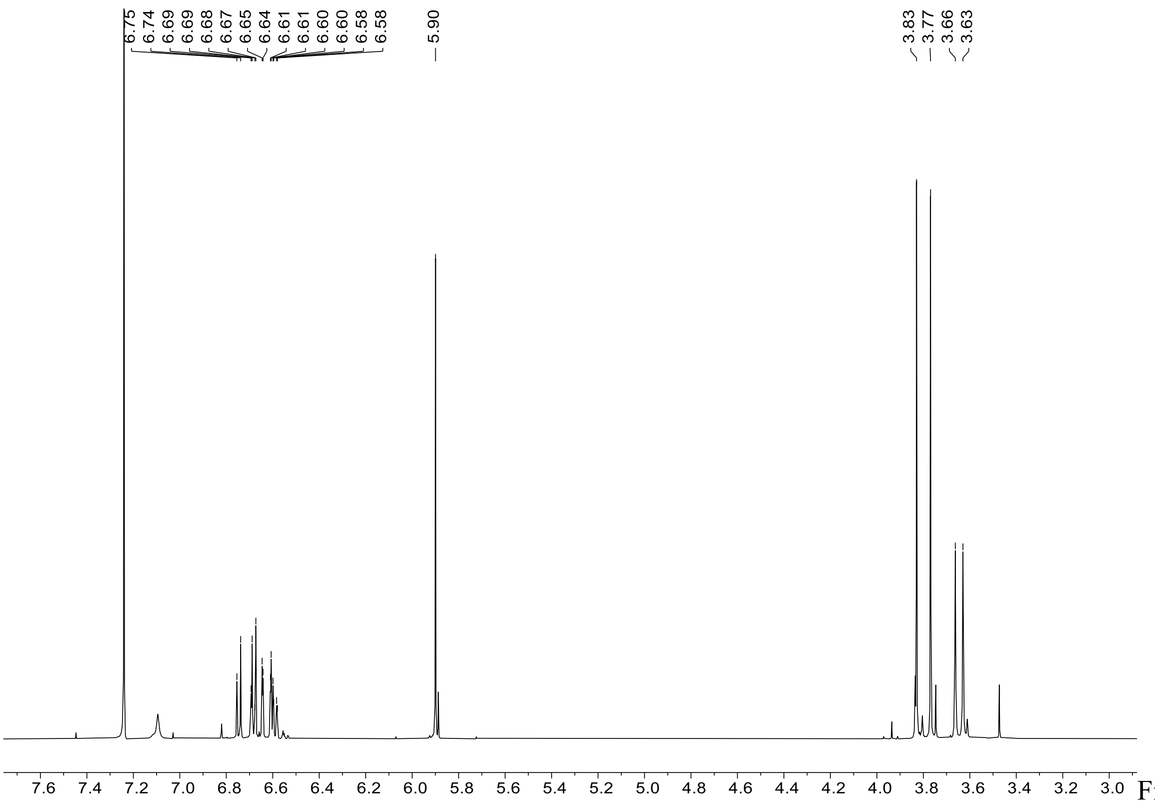

Figure S1. ^{1}H NMR spectrum ($CDCl_3$, 500 MHz) of **1**

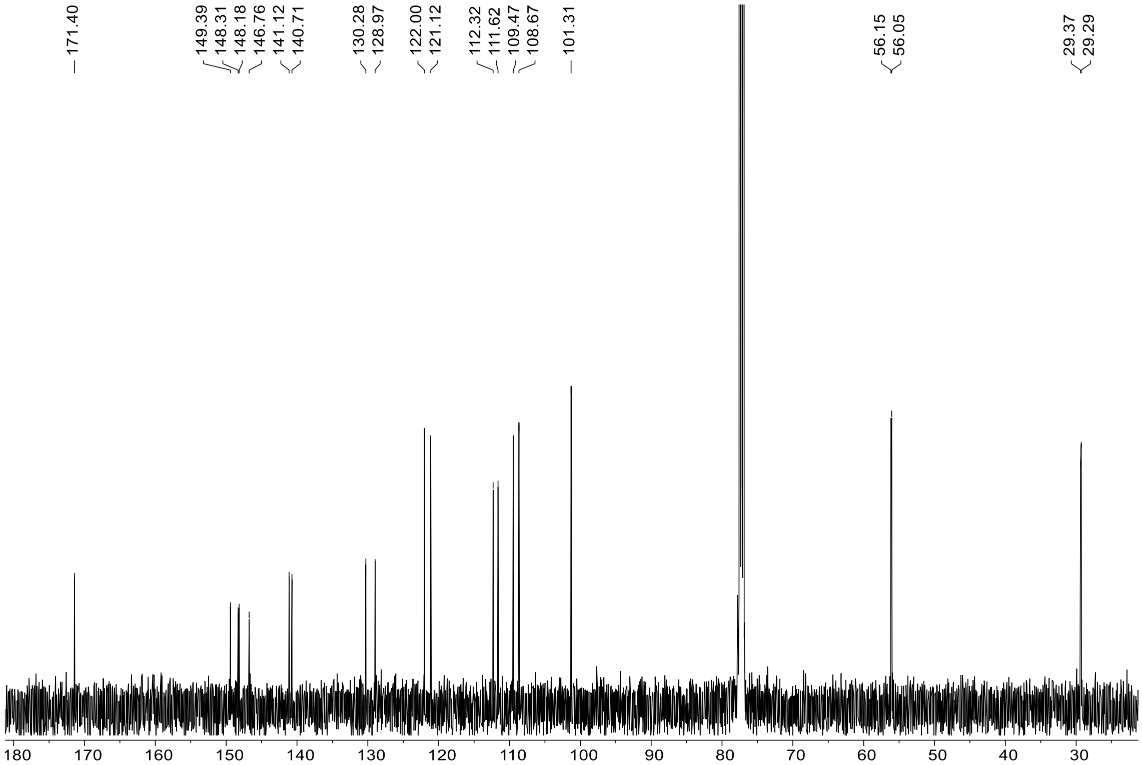

Figure S2. ^{13}C NMR spectrum ($CDCl_3$, 125 MHz) of **1**

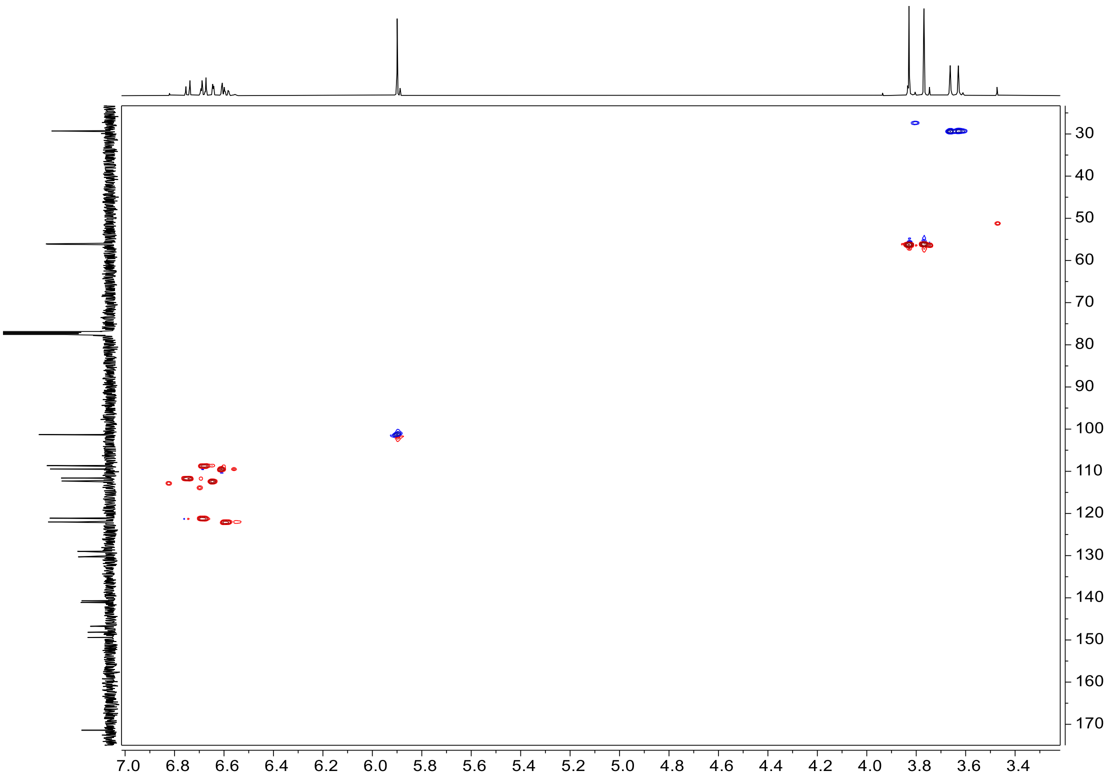

Figure S3. HSQC spectrum of **1**

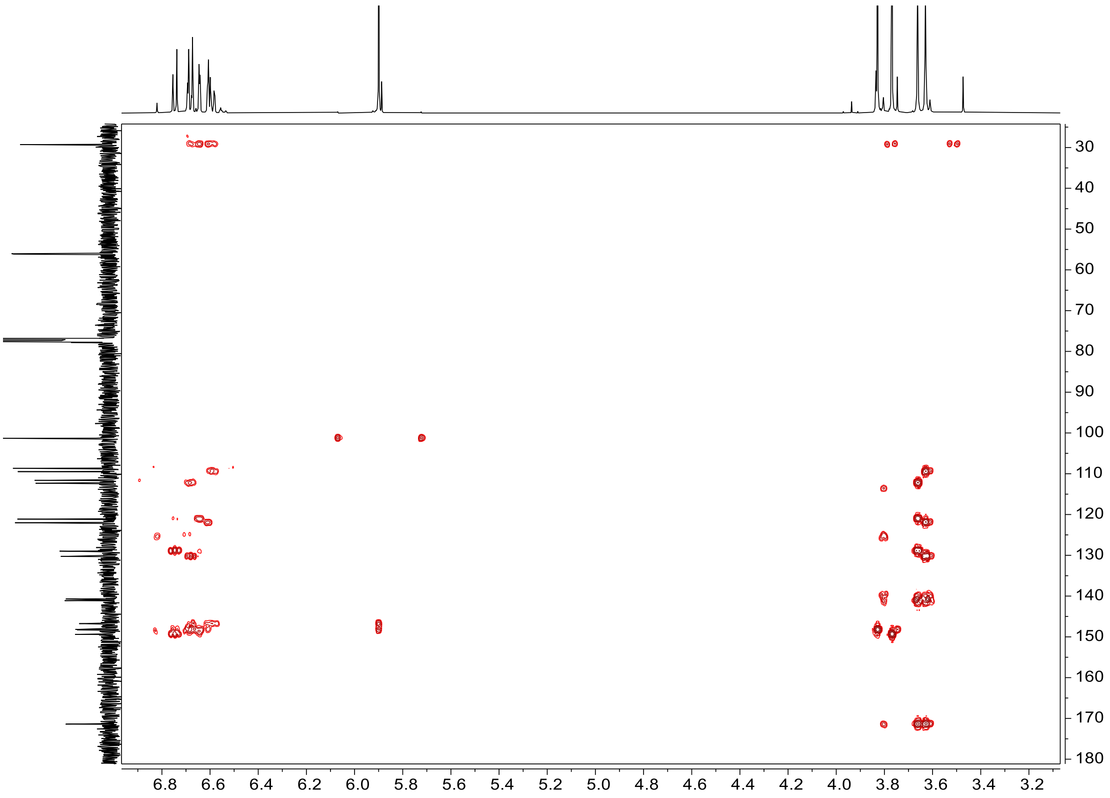

Figure S4. HMBC spectrum of **1**

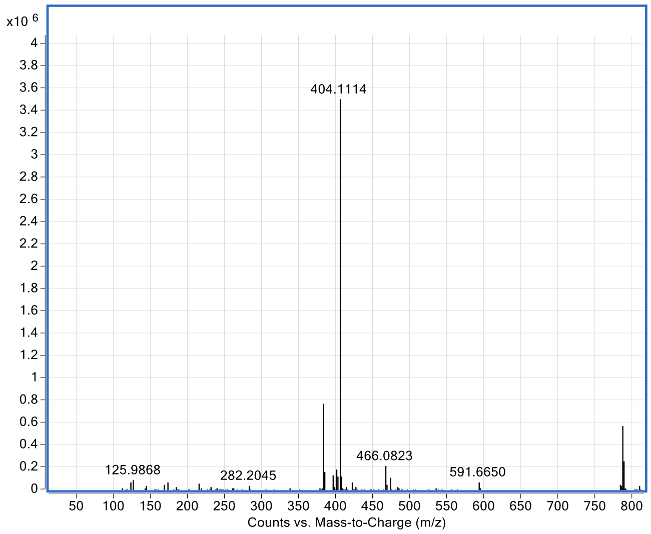

Figure S5. ESI-Q-TOF-MS spectrum of **1**

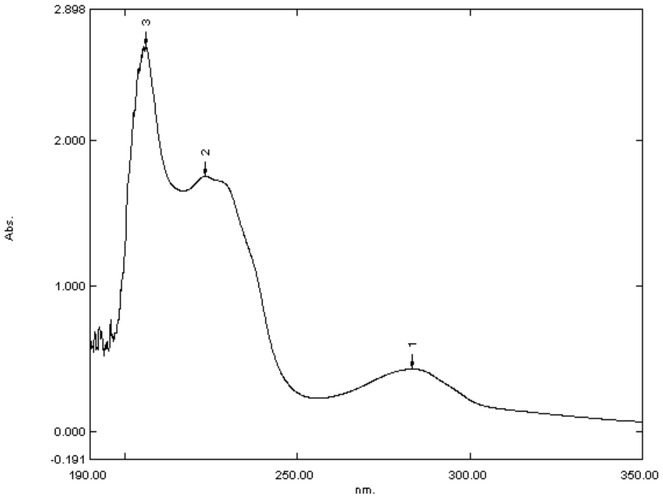

Figure S6. UV spectrum of **1**

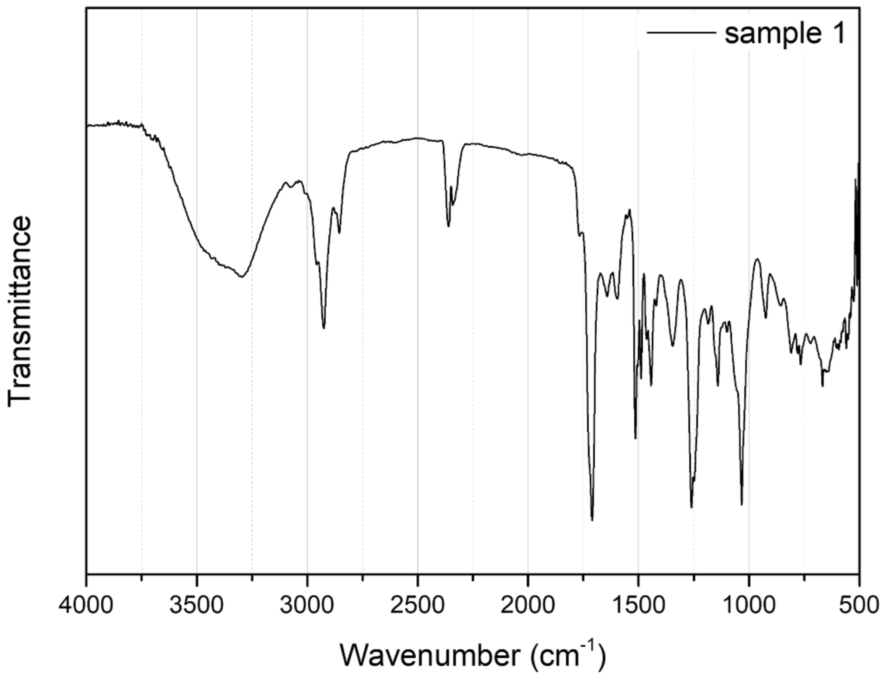

Figure S7. IR spectrum of **1**

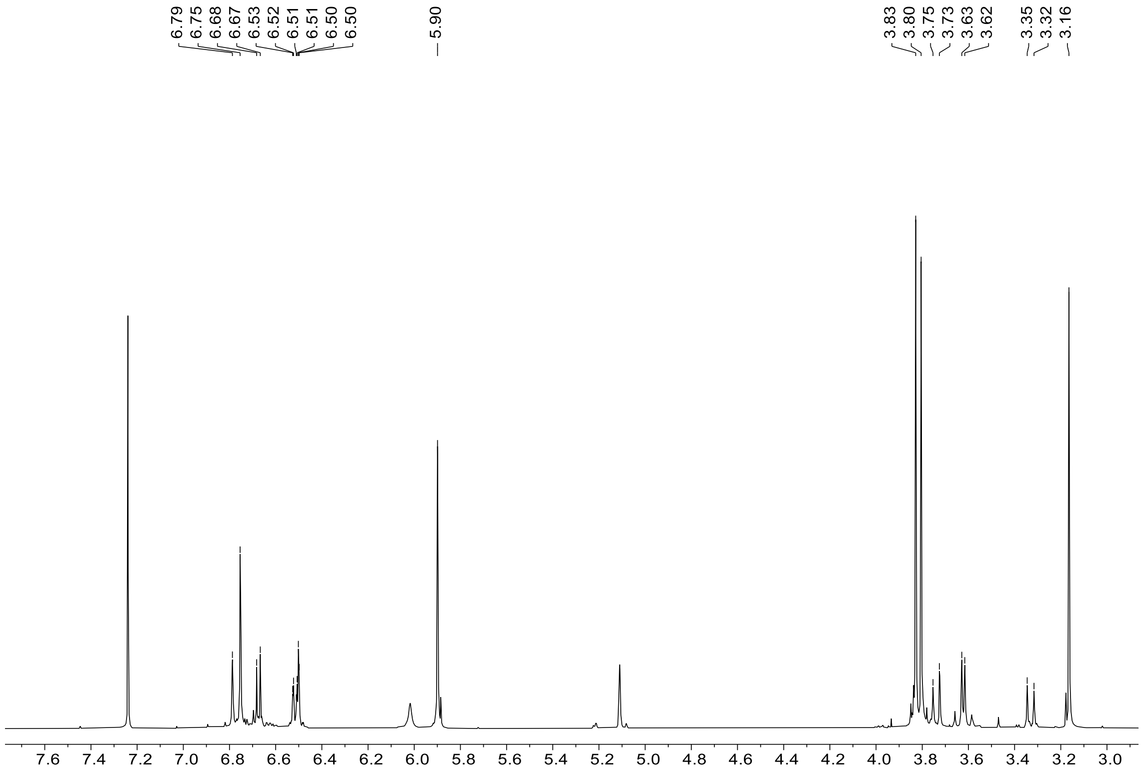

Figure S8. ¹H NMR spectrum ($CDCl_3$, 500 MHz) of **2**

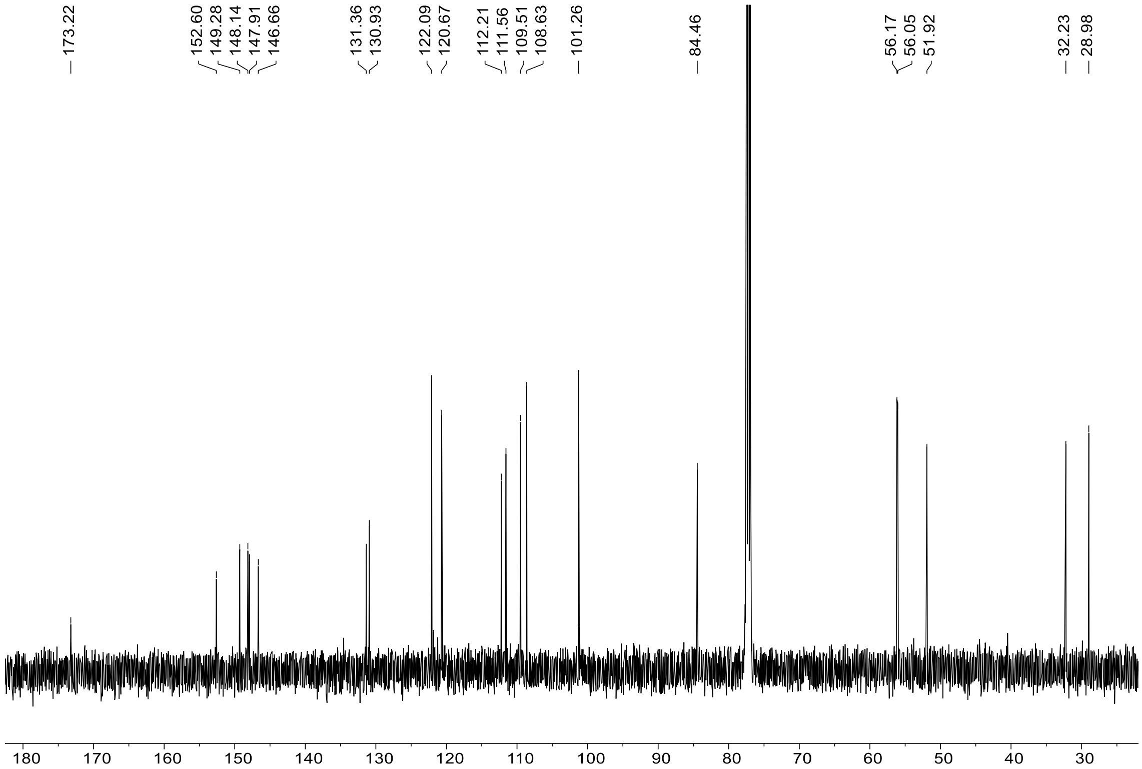

Figure S9. ¹³C NMR spectrum ($CDCl_3$, 125 MHz) of **2**

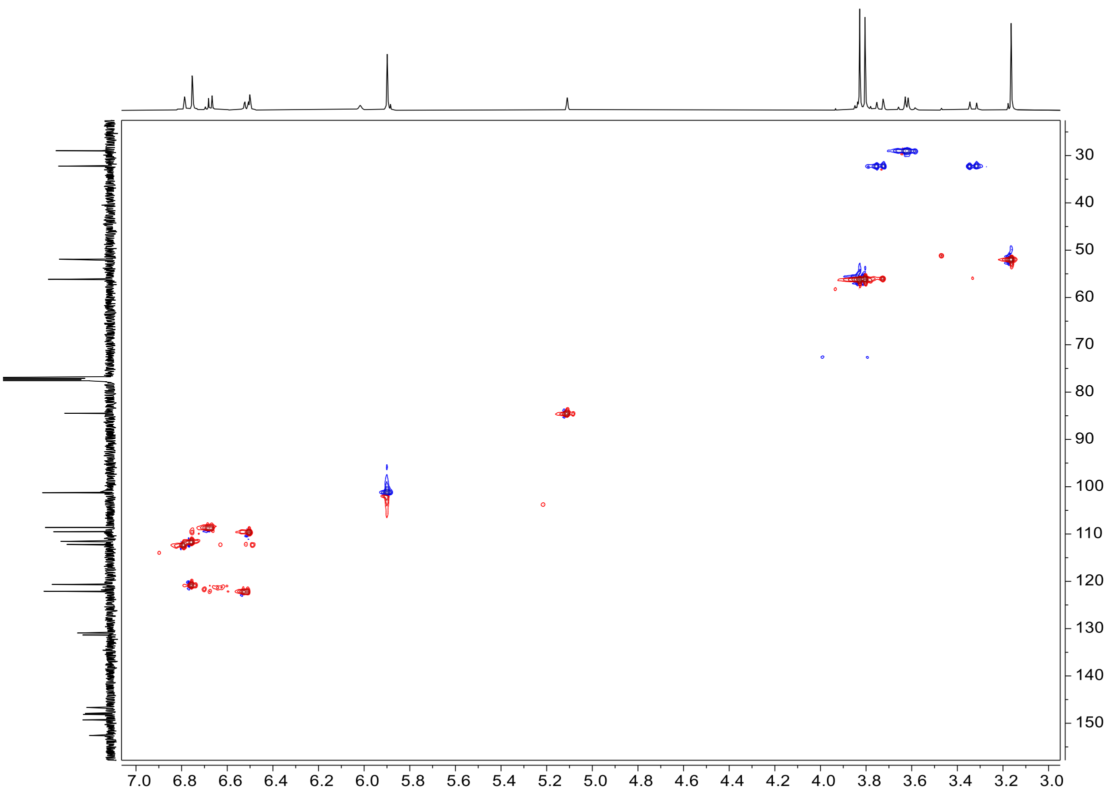

Figure S10. HSQC spectrum of **2**

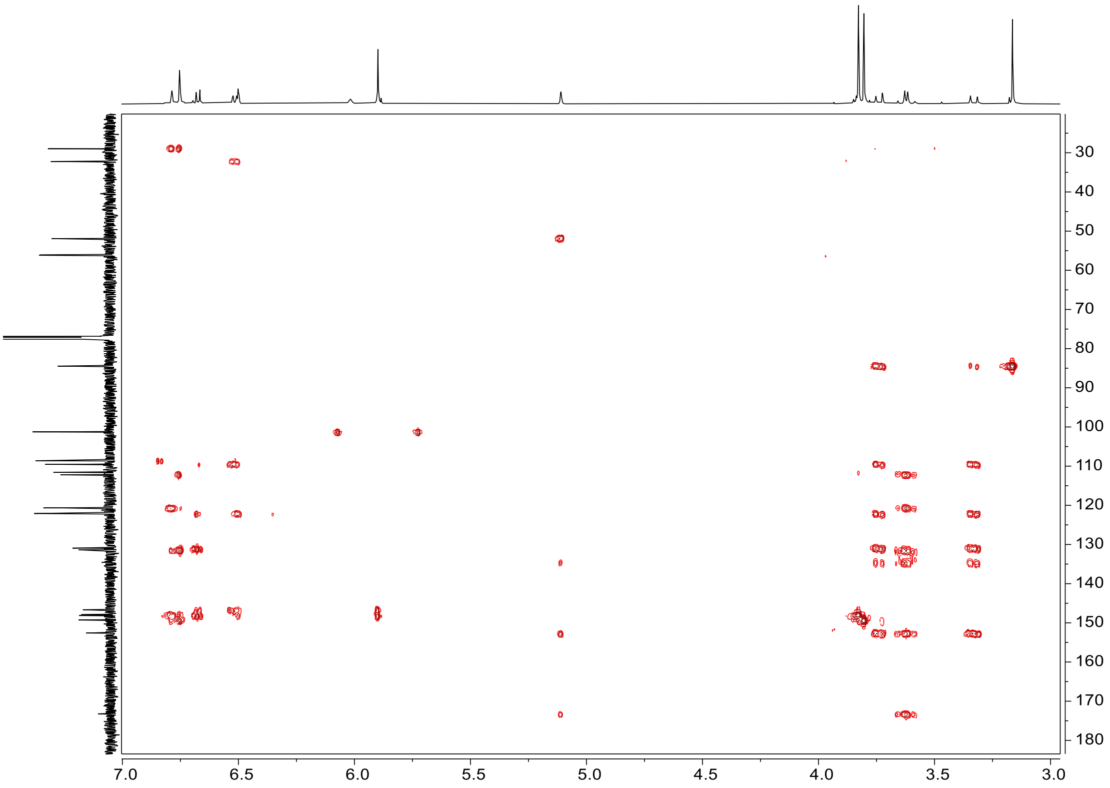

Figure S11. HMBC spectrum of **2**

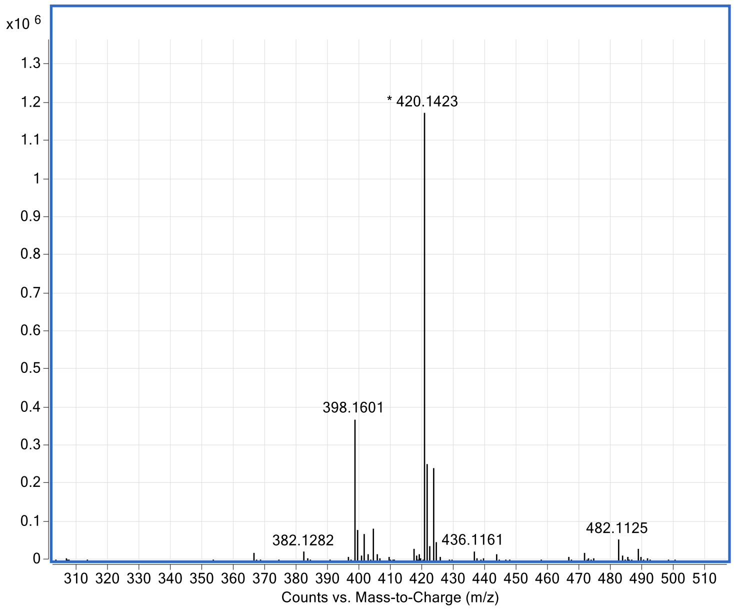

Figure S12. ESI-Q-TOF-MS spectrum of **2**

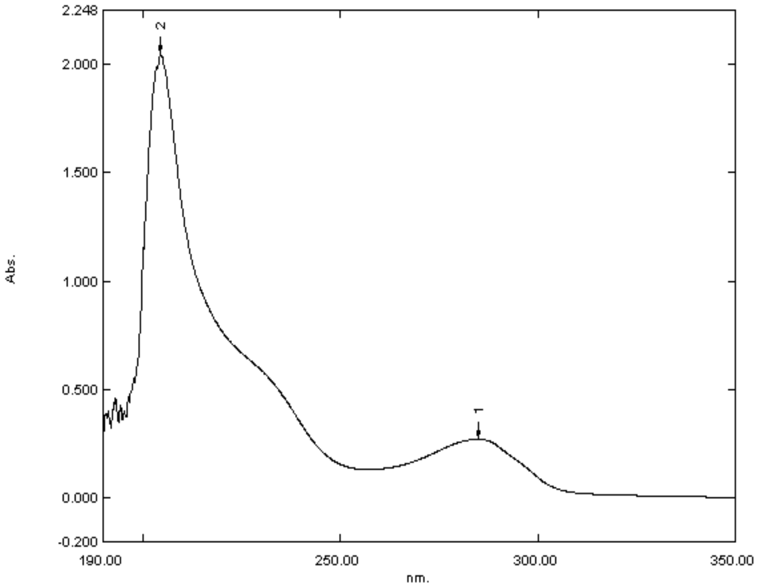

Figure S13. UV spectrum of **2**

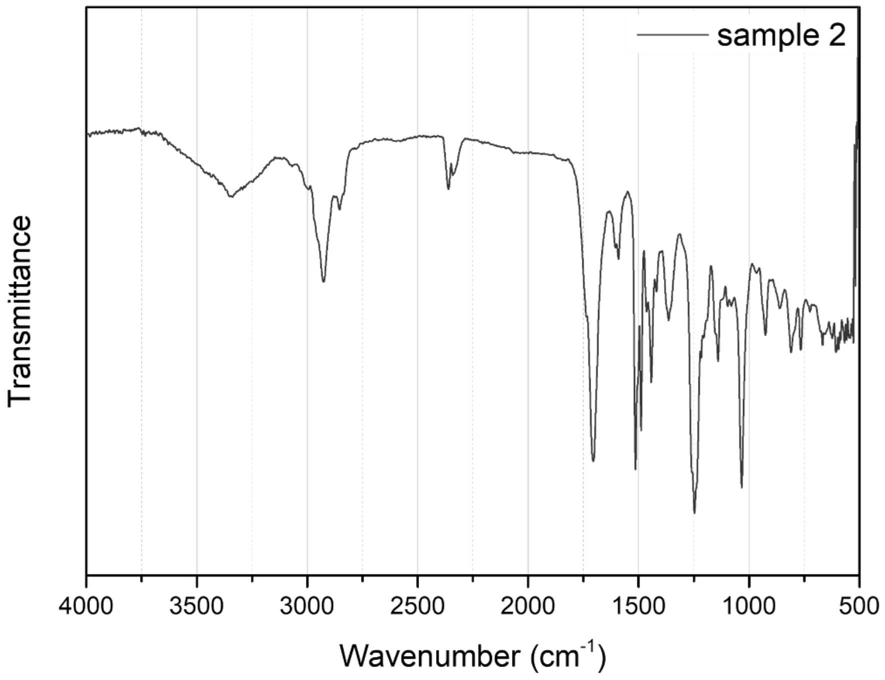

Figure S14. IR spectrum of **2**

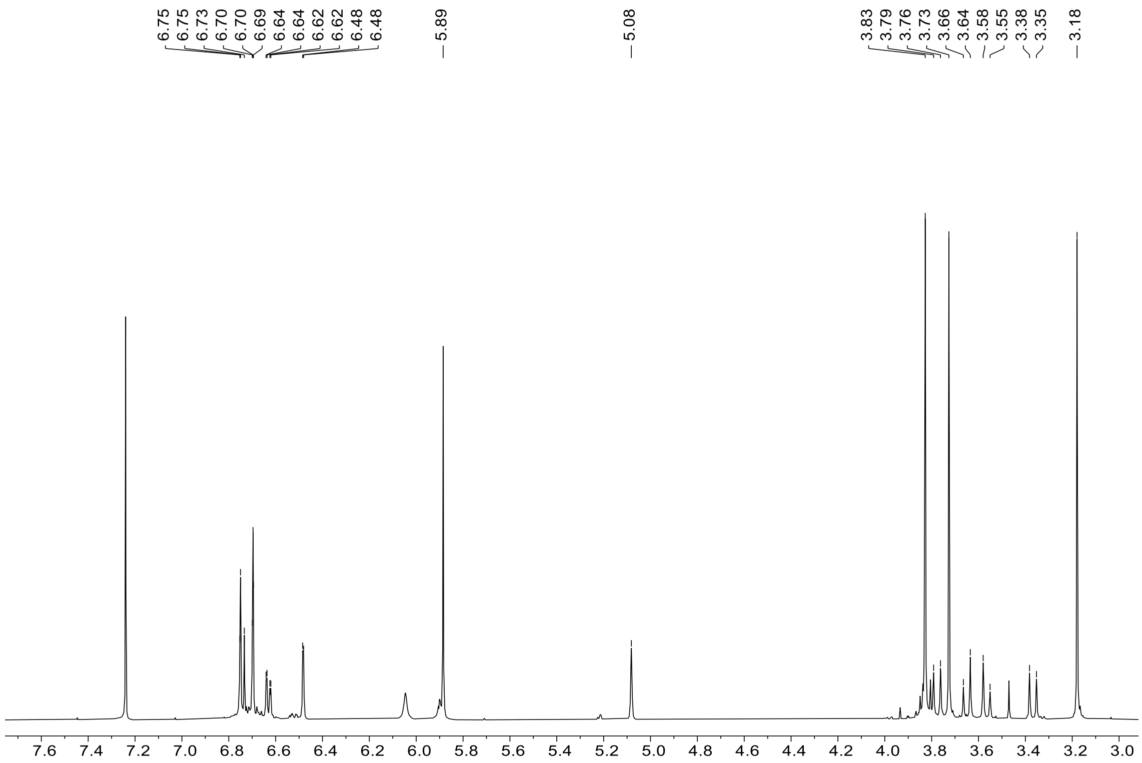

Figure S15. ^{1}H NMR spectrum ($CDCl_3$, 500 MHz) of **3**

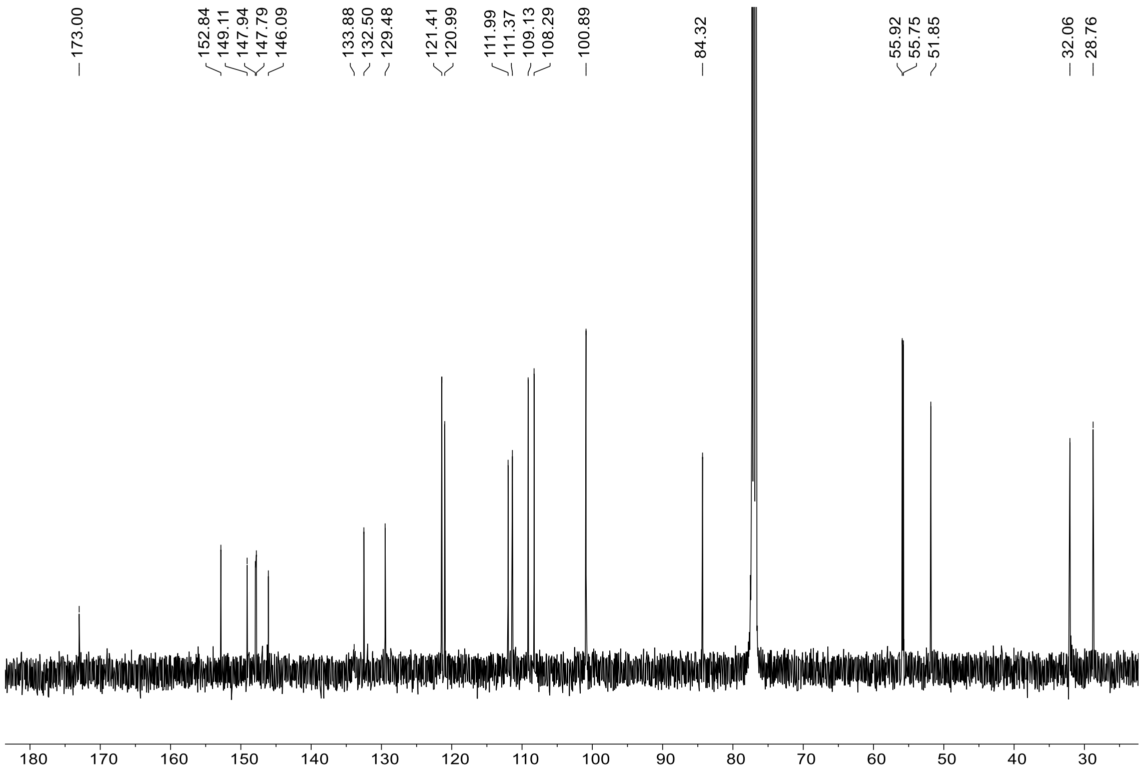

Figure S16. ^{13}C NMR spectrum ($CDCl_3$, 125 MHz) of **3**

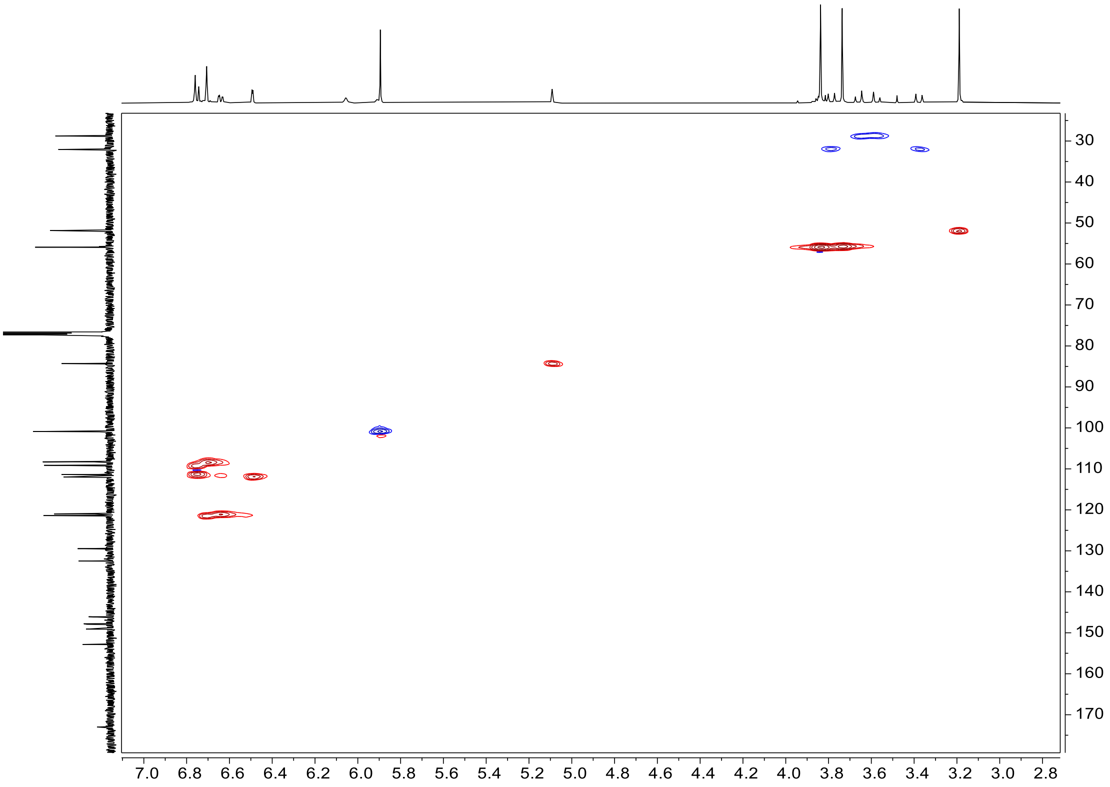

Figure S17. HSQC spectrum of **3**

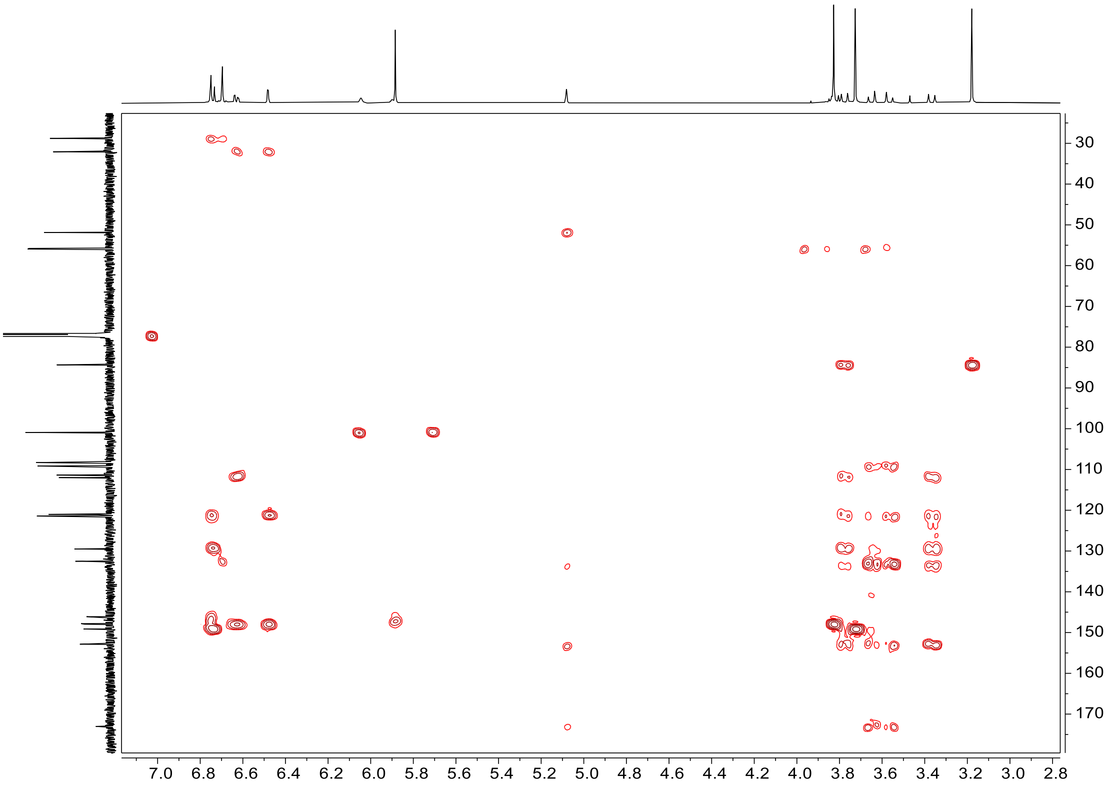

Figure S18. HMBC spectrum of **3**

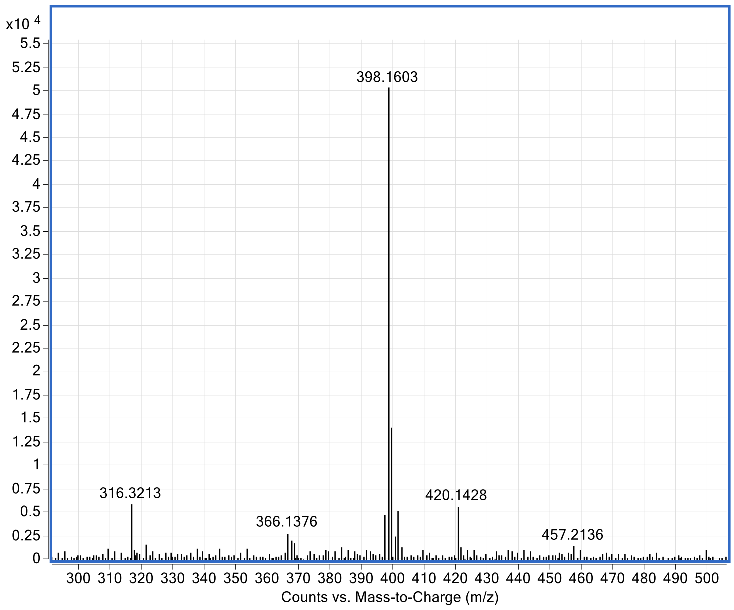

Figure S19. ESI-Q-TOF-MS spectrum of **3**

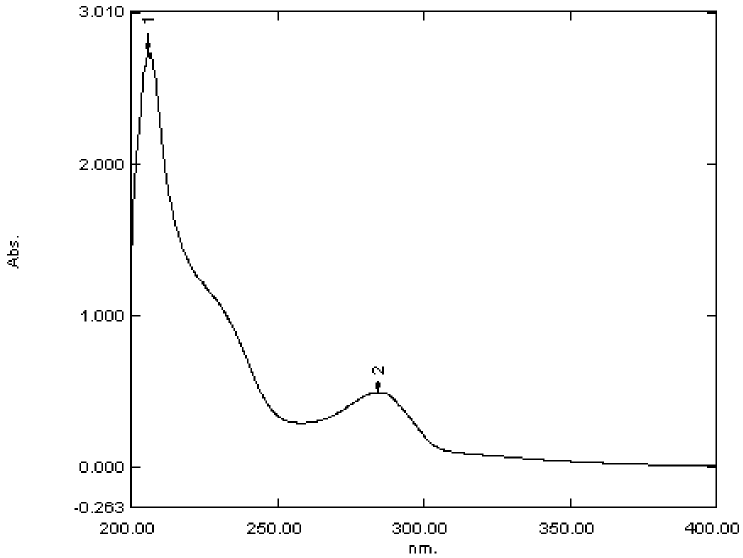

Figure S20. UV spectrum of **3**

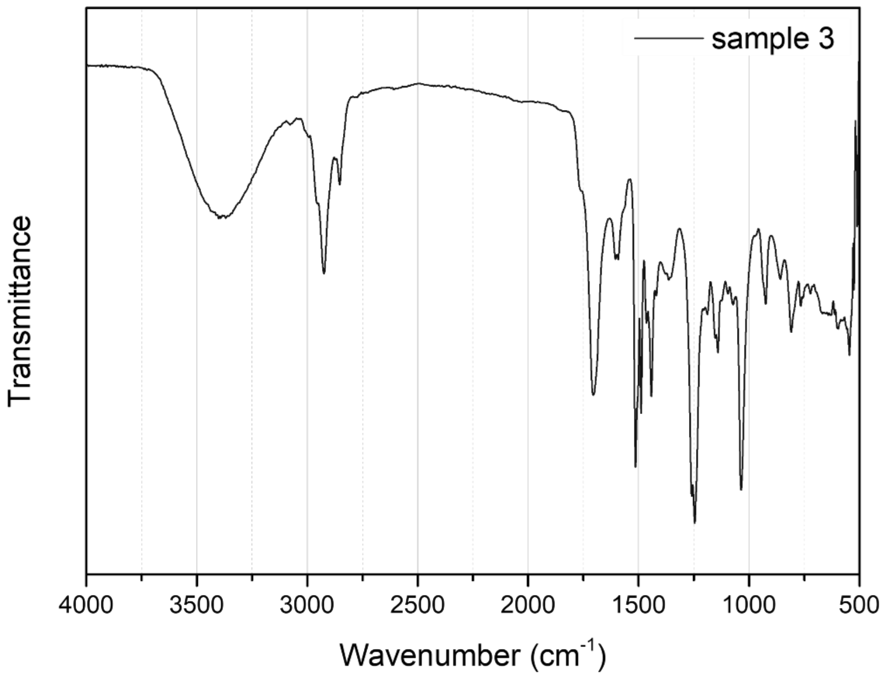

Figure S21. IR spectrum of **3**

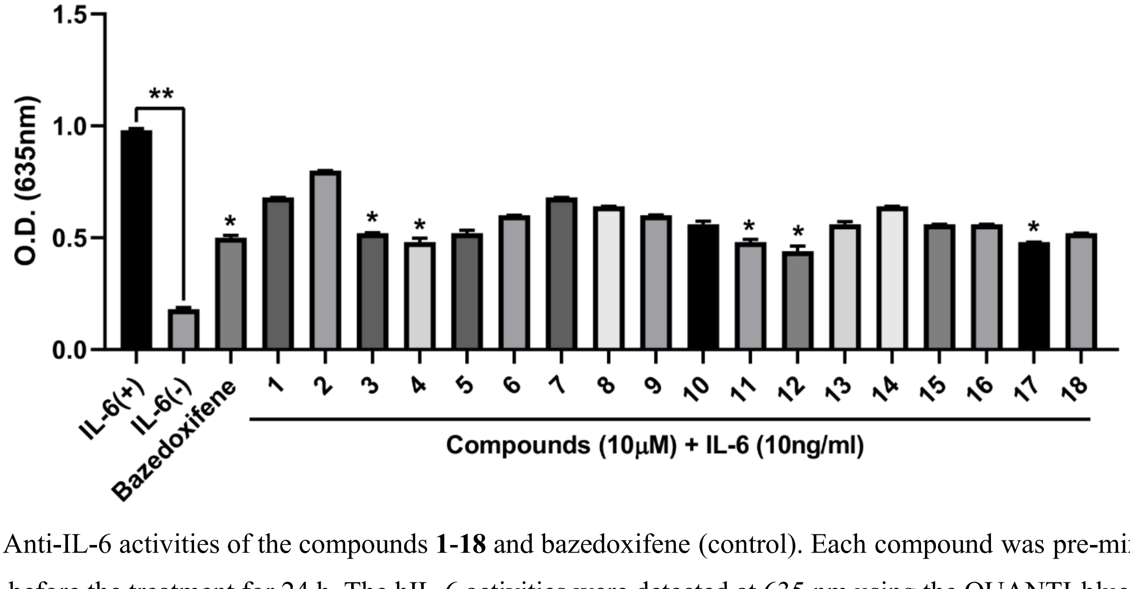

Figure S22. Anti-IL-6 activities of the compounds **1-18** and bazedoxifene (control). Each compound was pre-mixed with 10 ng/ml hIL-6 before the treatment for 24 h. The hIL-6 activities were detected at 635 nm using the QUANTI-blue assay. Data indicate mean ± SD (n = 3). *$p < 0.05$ and ** $p < 0.01$ vs. IL-6 (+)

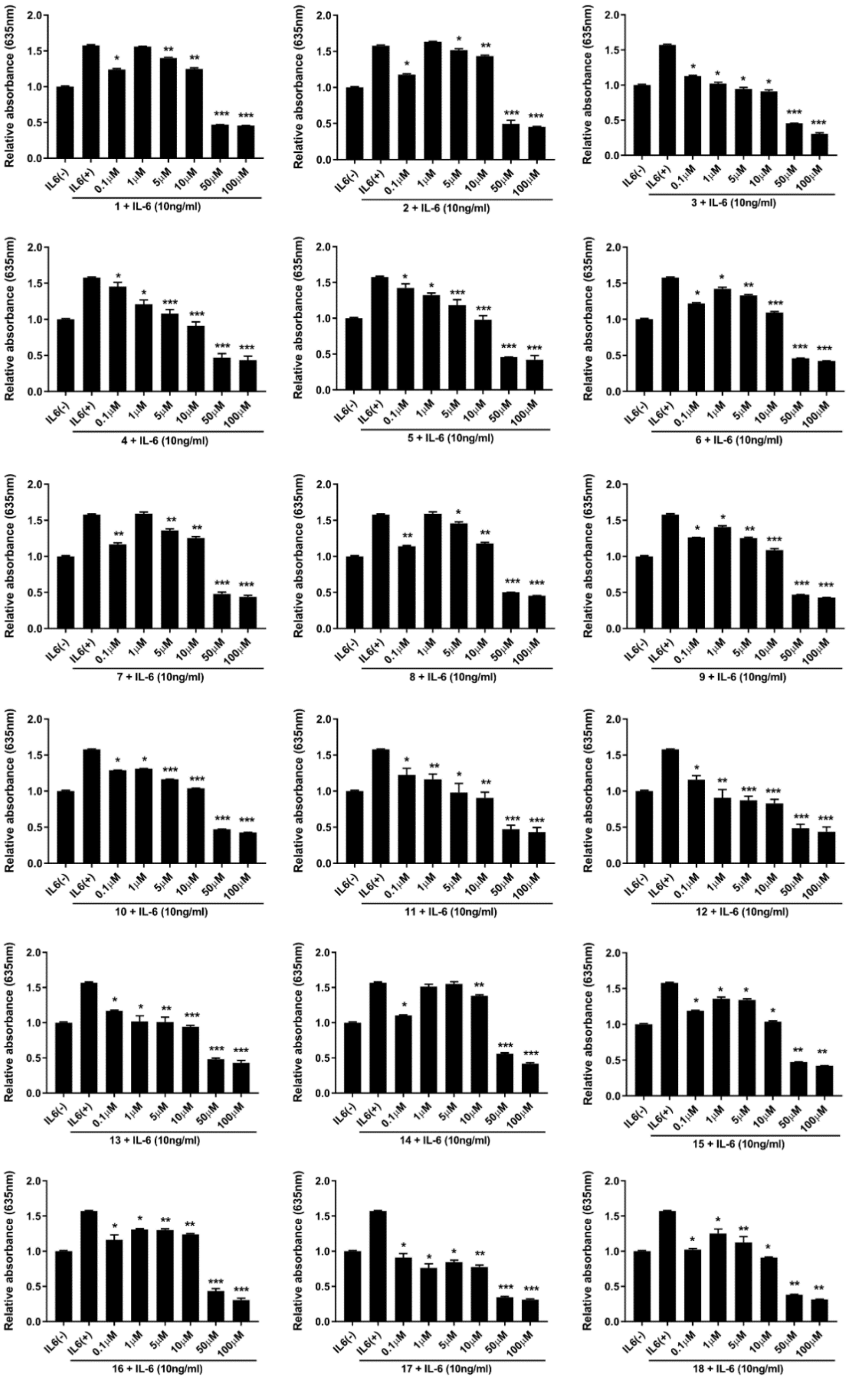

Figure S23. **Bioactivity test with eighteen compounds in HEK-Blue™ IL-6 cells.** HEK-Blue™ IL-6 cells were treated with various concentration of compounds 1-18 for 24 h. The activation of IL-6 was measured by a SEAP activity assay after the treatment of HEK-Blue™ IL-6 cells with the different indicated concentrations of compounds 1-18 for 30 min in the presence or absence of IL-6 for 24 h. Data indicate mean ± SD (n = 3). **p*<0.05, ***p*<0.01 and ****p*<0.001 vs. IL-6(+).

Table S1. HPLC chromatogram of compounds 1 – 18

Table S1. Analytical HPLC condition for compounds **1–18**.

<table>
<tr><td>Instrument</td><td>Agilent Technologies 1260 Infinity</td></tr>
<tr><td>HPLC column</td><td>Luna C18(2) column (250 × 4.6 mm, 5 µm; Phenomenex)</td></tr>
<tr><td>Detection wavelength</td><td>254 nm for compounds 1–11 and 13–18
203 nm for compound 12</td></tr>
<tr><td>Flow rate</td><td>1.0 mL/min</td></tr>
<tr><td>Injection volume</td><td>20 µL</td></tr>
<tr><td>Mobile phase condition</td><td><table><tr><td>Time (min)</td><td>Water (0.01 % TFA)</td><td>MeCN (0.01 % TFA)</td></tr><tr><td>0</td><td>95 %</td><td>5 %</td></tr><tr><td>5</td><td>95 %</td><td>5 %</td></tr><tr><td>50</td><td>0</td><td>100 %</td></tr></table></td></tr>
</table>

Table S2. HPLC chromatogram for compounds **1–18**.

#	HPLC chromatogram	Relative Peak Area (%)
**1**	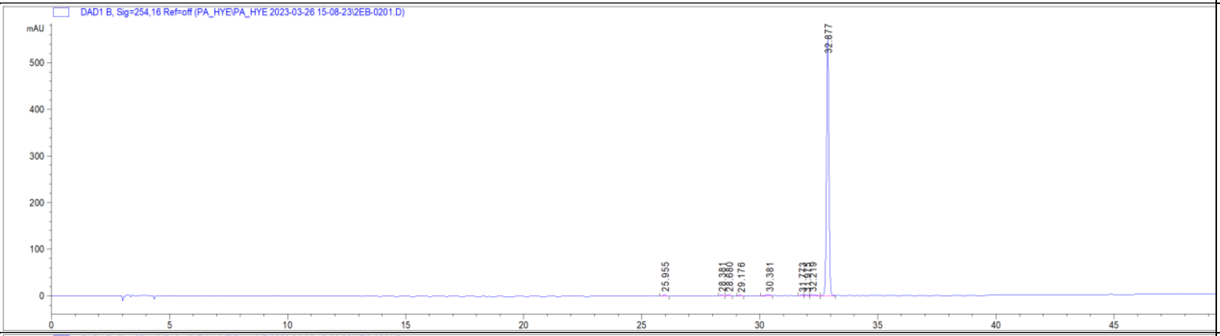	95.3
**2**	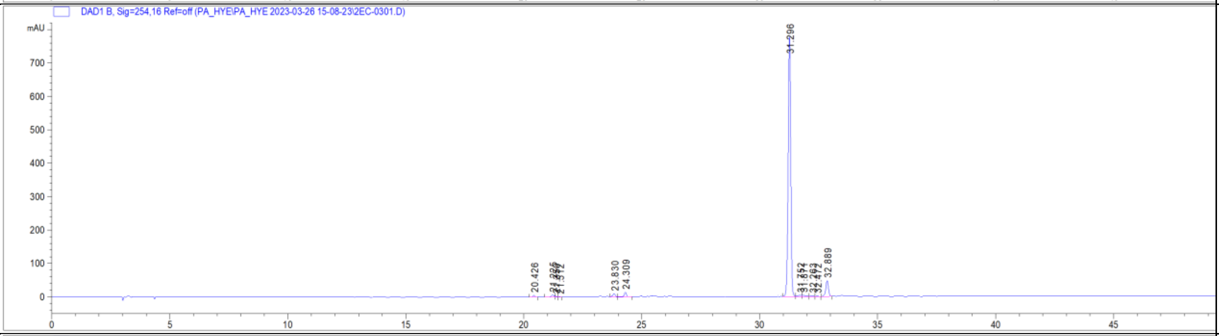	88.9
**3**	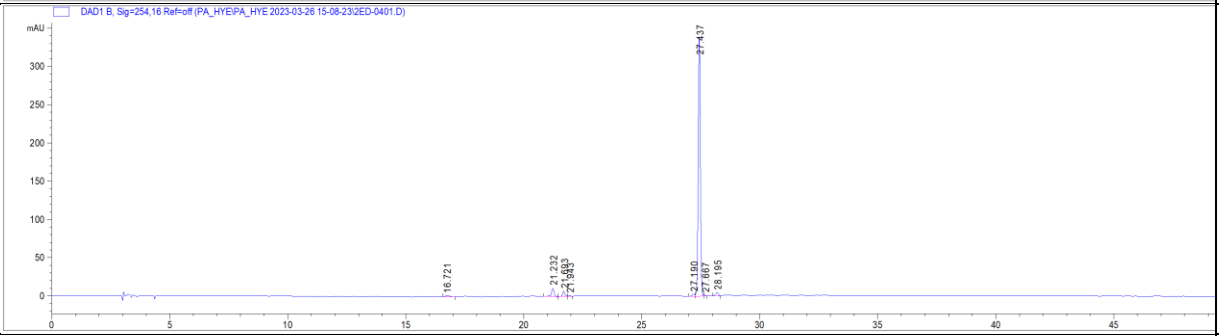	90.8
**4**	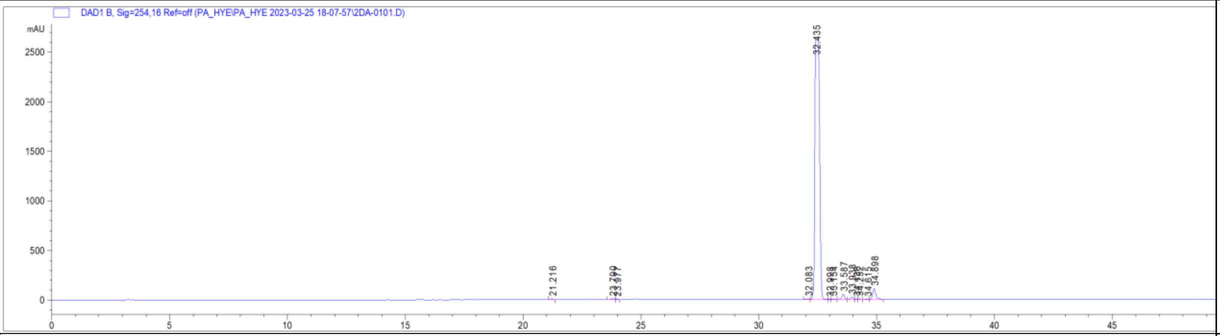	93.6
**5**	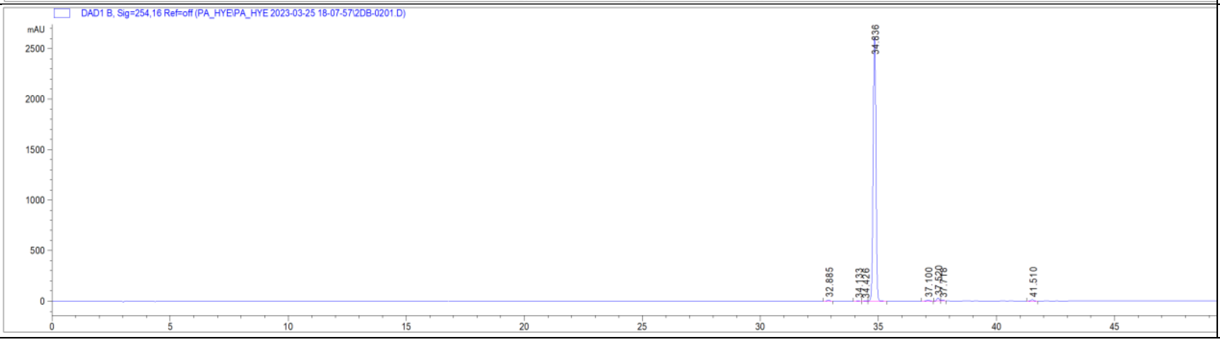	97.1

#	HPLC chromatogram	Relative Peak Area (%)
**6**	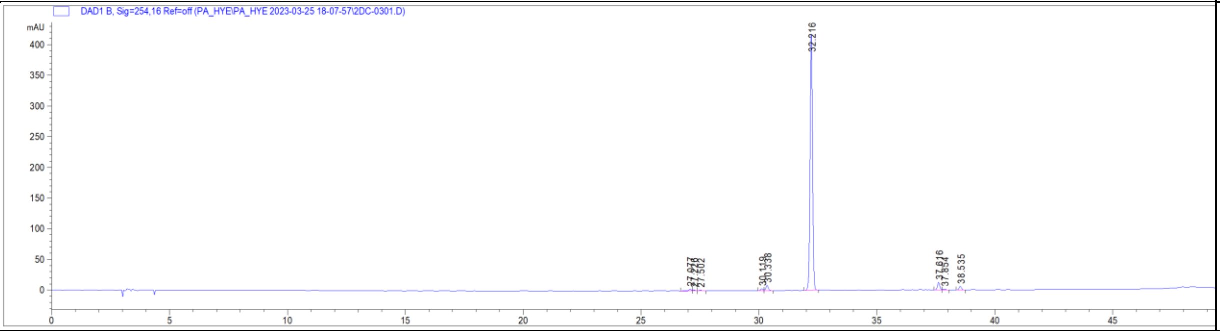	91.8
**7**	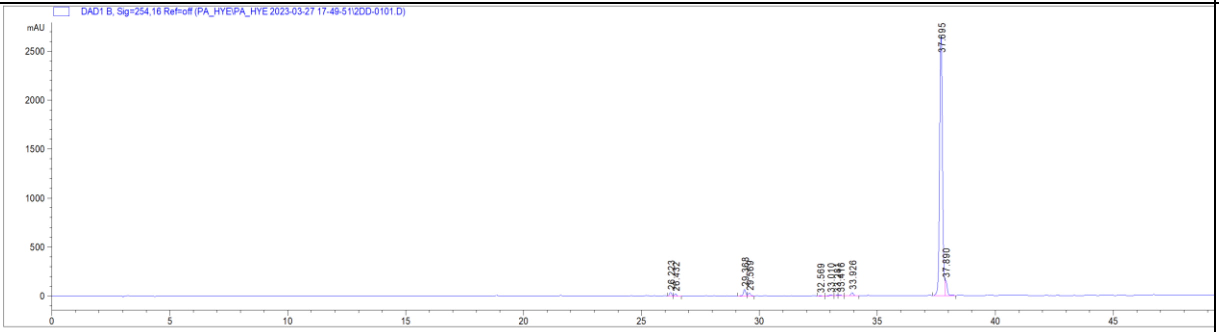	90.2
**8**	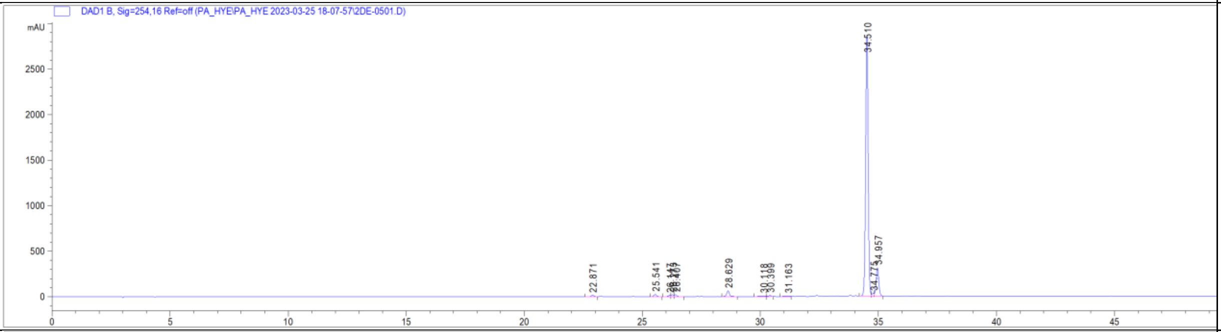	84.0
**9**	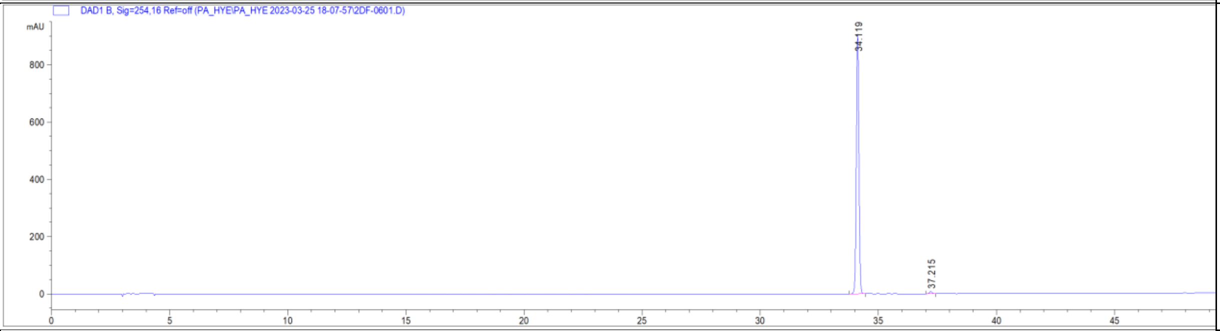	99.1
**10**	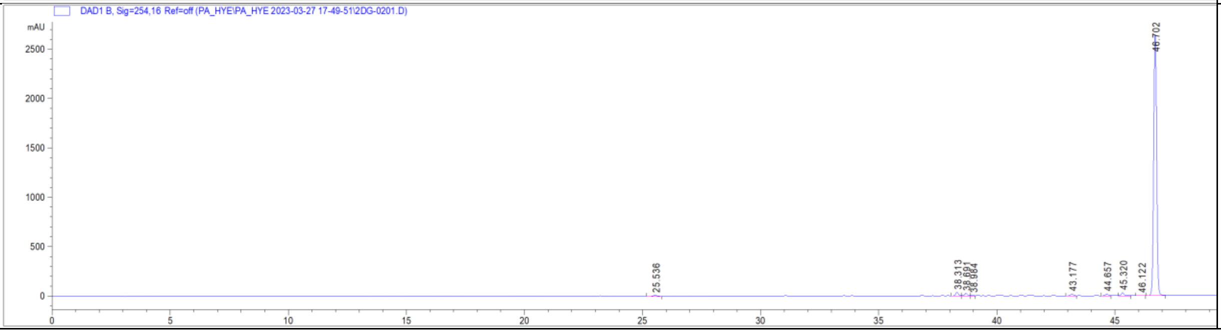	95.0

#	HPLC chromatogram	Relative Peak Area (%)
**11**	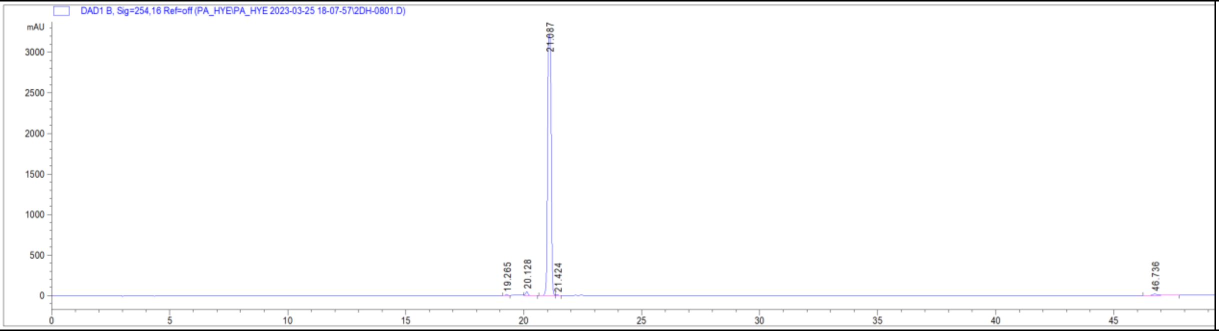	97.7
**12**	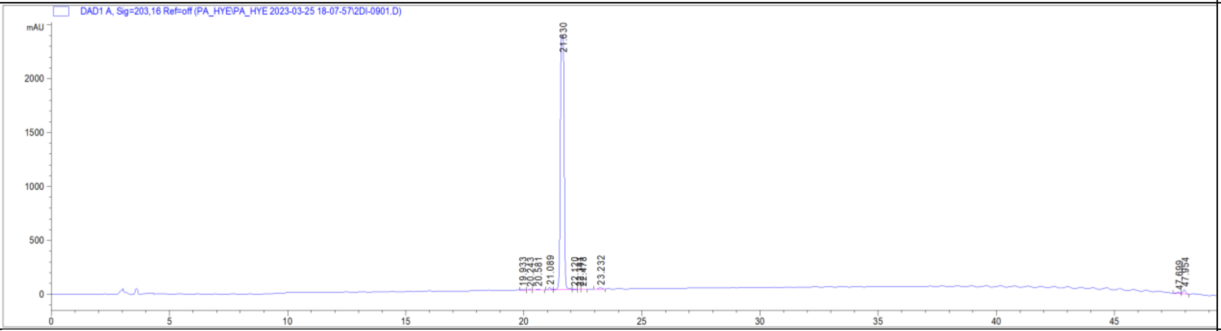	96.2
**13**	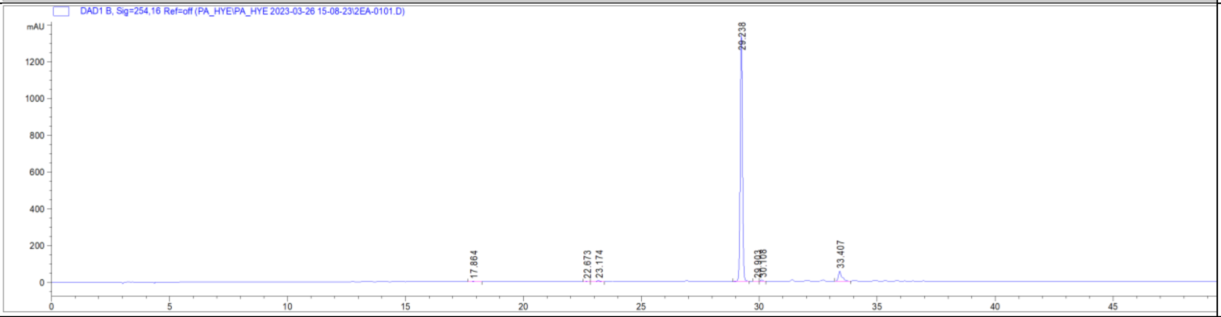	92.6
**14**	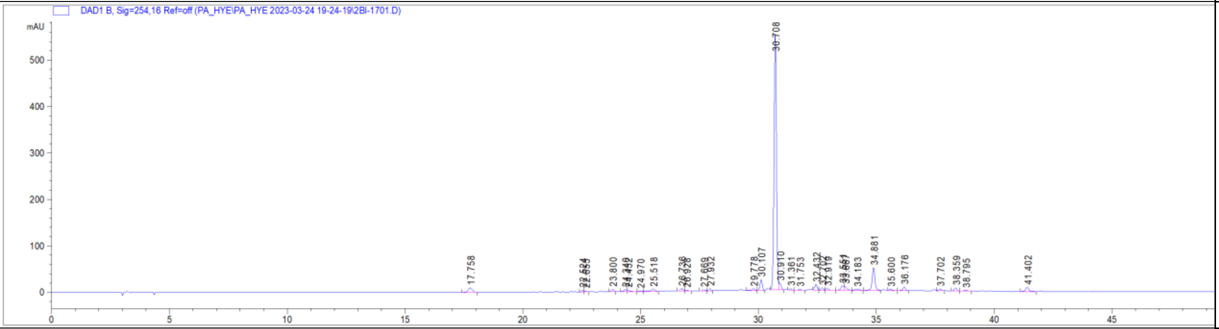	68.2
**15**	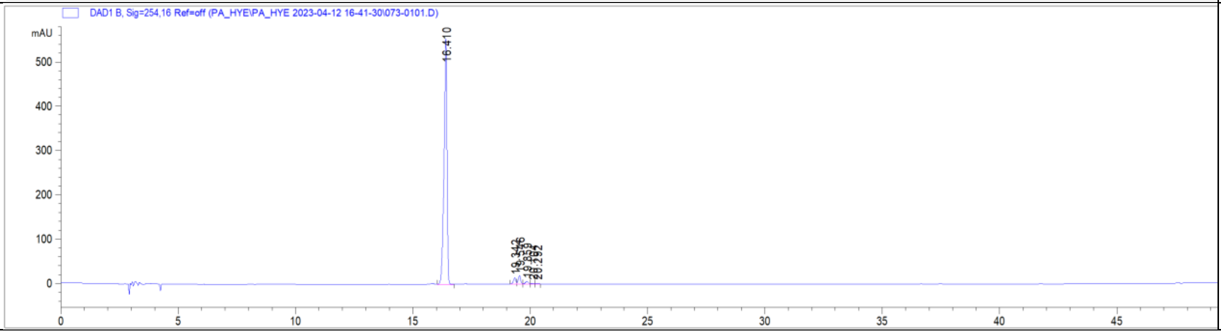	94.5

#	HPLC chromatogram	Relative Peak Area (%)
**16**	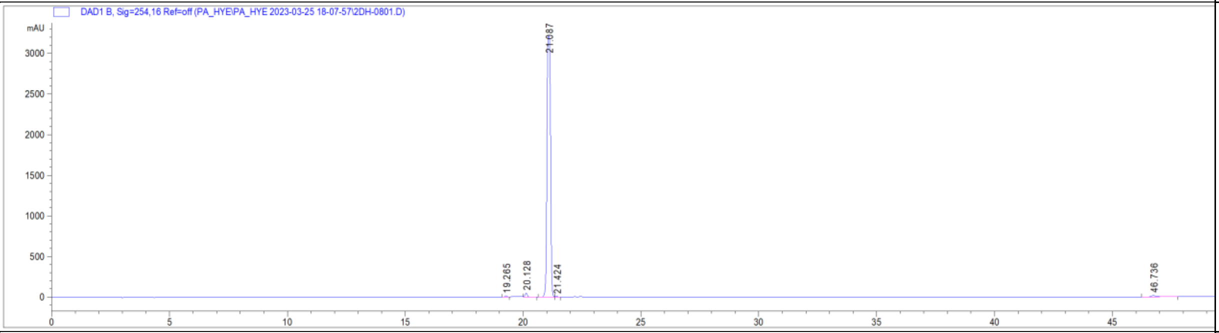	95.1
**17**	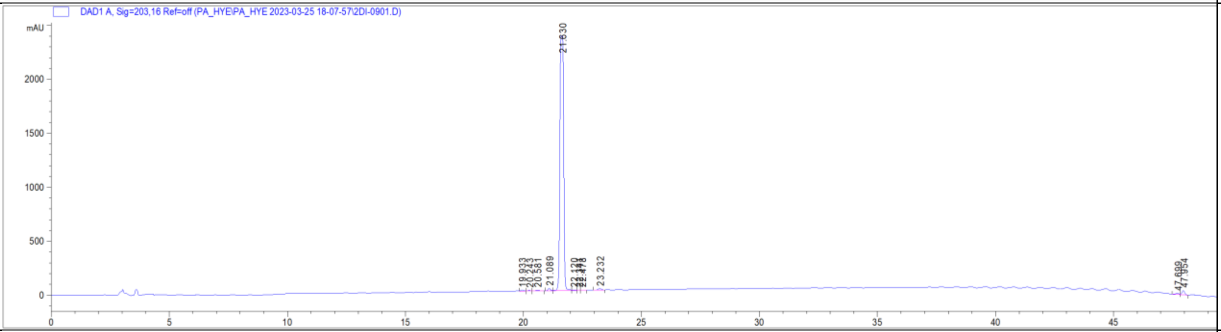	90.9
**18**	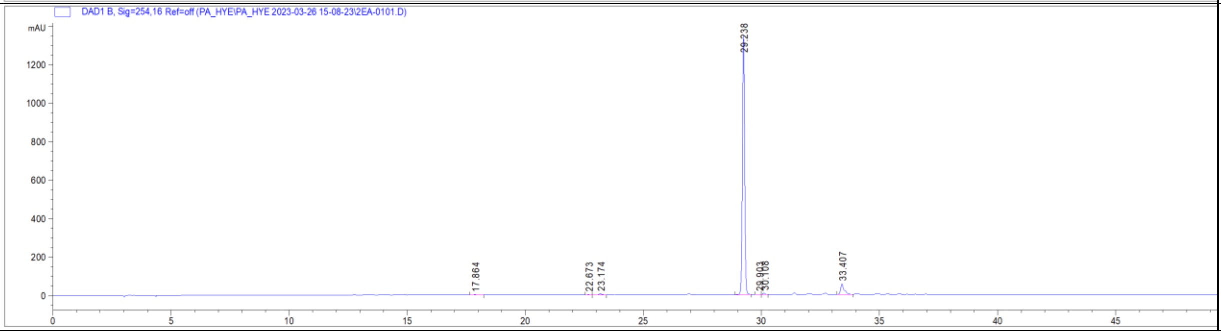	85.6
